# Links between DNA methylation and nucleosome occupancy in the human genome

**DOI:** 10.1186/s13072-017-0125-5

**Published:** 2017-04-11

**Authors:** Clayton K. Collings, John N. Anderson

**Affiliations:** 1 Department of Biochemistry and Molecular Genetics, Northwestern University Feinberg School of Medicine, 320 E. Superior Street, Chicago, IL 60611 USA; 2 Department of Biological Sciences, Purdue University, 915 W. State Street, West Lafayette, IN 47907 USA

**Keywords:** DNA methylation, Nucleosome, Epigenetics, CpG, NOMe-seq, MNase-seq

## Abstract

**Background:**

DNA methylation is an epigenetic modification that is enriched in heterochromatin but depleted at active promoters and enhancers. However, the debate on whether or not DNA methylation is a reliable indicator of high nucleosome occupancy has not been settled. For example, the methylation levels of DNA flanking CTCF sites are higher in linker DNA than in nucleosomal DNA, while other studies have shown that the nucleosome core is the preferred site of methylation. In this study, we make progress toward understanding these conflicting phenomena by implementing a bioinformatics approach that combines MNase-seq and NOMe-seq data and by comprehensively profiling DNA methylation and nucleosome occupancy throughout the human genome.

**Results:**

The results demonstrated that increasing methylated CpG density is correlated with nucleosome occupancy in the total genome and within nearly all subgenomic regions. Features with elevated methylated CpG density such as exons, SINE-Alu sequences, H3K36-trimethylated peaks, and methylated CpG islands are among the highest nucleosome occupied elements in the genome, while some of the lowest occupancies are displayed by unmethylated CpG islands and unmethylated transcription factor binding sites. Additionally, outside of CpG islands, the density of CpGs within nucleosomes was shown to be important for the nucleosomal location of DNA methylation with low CpG frequencies favoring linker methylation and high CpG frequencies favoring core particle methylation. Prominent exceptions to the correlations between methylated CpG density and nucleosome occupancy include CpG islands marked by H3K27me3 and CpG-poor heterochromatin marked by H3K9me3, and these modifications, along with DNA methylation, distinguish the major silencing mechanisms of the human epigenome.

**Conclusions:**

Thus, the relationship between DNA methylation and nucleosome occupancy is influenced by the density of methylated CpG dinucleotides and by other epigenomic components in chromatin.

**Electronic supplementary material:**

The online version of this article (doi:10.1186/s13072-017-0125-5) contains supplementary material, which is available to authorized users.

## Background

The genomes of eukaryotic organisms are packaged into tightly condensed arrangements of nucleoprotein complexes referred to as chromatin. At the primary level of chromatin compaction, a 147-base-pair segment of DNA spirals nearly twice around an octamer of histone proteins to form a structure known as the nucleosome [1, 2]. The degree of nucleosome occupancy that occurs along DNA in chromatin is important because it can dictate the accessibility of DNA to the transcriptional machinery and to other proteins involved in genome regulation [3]. With advances in high-throughput sequencing technologies, nucleosome maps have revealed differential nucleosome occupancy patterns over entire genomes for a variety of species and cell types [4]. For example, nucleosome-depleted regions are observed overlapping transcription start sites of active genes, while high nucleosome occupancy is found to encompass the promoters of silent genes [5]. Furthermore, several genome‐wide explorations in conjunction with biochemical modifications have elucidated mechanisms that have been evoked to explain the differences in nucleosome occupancy detected across intragenic and intergenic chromatin.

In vertebrate cells, the most common mode of DNA methylation entails the addition of a methyl group to a cytosine residue in the context of a CpG dinucleotide. CpG methylation is perhaps the best understood epigenetic mark and is maintained through cell division during DNA replication primarily by the DNA methyltransferase Dnmt1 with some assistance from the de novo methyltransferases, Dnmt3a and Dnmt3b [6]. This modification has been linked to gene silencing and is considered to be an important factor in the formation of constitutive and facultative heterochromatin [7]. Additionally, DNA methylation has also been shown to be essential for normal tissue-specific development. During embryonic stem cell differentiation, select CpGs throughout the genome become methylated by the de novo DNA methyltransferases, and through DNA methylation’s epigenetic influence on chromatin structure and gene regulation, the inheritability of diverse cellular phenotypes within higher eukaryotic species is sustained [8, 9].

Although CpG dinucleotides are underrepresented in mammals, they are not randomly distributed, and regions with high CpG density, referred to as CpG islands, are typically unmethylated and cover the promoters of many housekeeping genes [10]. The chromatin architecture of active CpG island promoters is characterized by nucleosome depletion, histone acetylation, H3K4 methylation, but not H3K36 methylation [10]. Additionally, numerous transcription factors bind to CpG islands, and proteins with CXXC domains, which target unmethylated CpGs, are especially enriched in CpG Islands [10]. Some examples include the transcription factor Sp1 [11], the H3K36me2 demethylase Kdm2a [12], and the H3K4me3 methyltransferase subunit Cfp1 [13]. These features of active transcription are thought to protect CpG islands from de novo methylation [10].

Downstream of active promoters, Setd2 catalyzes the methylation of H3K36 with elongating Pol II in the bodies of transcribed genes [5]. H3K36me3 is enriched in exons over introns and has been proposed to be associated with co-transcriptional splicing mechanisms [14]. With the transfer of Pol II, histone H3K4 demethylation is performed by Kdm5 and Kdm1 [15]. In parallel, the de novo DNA methyltransferases preferentially bind to unmethylated H3K4 and to H3K36me3 [16–19], and recently, the presence of H3K36me3 was shown to be linked to the enrichment of binding and de novo methylation by DNMT3b over DNMT3a in gene bodies in vivo [20].

We previously examined the effects of DNA methylation on the stability of a large heterogeneous population of nucleosomes [21]. Specifically, with bacterial artificial chromosomes (BACs), the CpG methyltransferase M. SssI, isolated histones, and micrococcal nuclease, we conducted nucleosome reconstitution experiments in conjunction with high‐throughput sequencing on ~1 MB of mammalian DNA that was unmethylated or methylated. The features by which DNA methylation was found to increase the stability of nucleosomes (already positioned by nucleotide sequence) were elevated CpG frequency and a tendency for the minor grooves of CpGs to be rotationally oriented toward the histone surface, and these methylation‐sensitive nucleosomes were found to be enriched in exons and in CpG islands [21]. Our in vitro nucleosome data reflected nucleosomal DNA methylation patterns observed in vivo in terms of the co‐enrichment of DNA methylation and nucleosome occupancy in exons [22–25], the increased nucleosome occupancy associated with methylated CpG islands [25, 26], and the rotational orientation of methylated CpGs in Arabidopsis nucleosomes [22].

In order to extend our research beyond our in vitro experiments, we sought to understand the relationship between DNA methylation and nucleosome occupancy in the cell. In this study, we first perform an integrated analysis of MNase-seq and NOMe-seq data. Through this approach, we survey chromatin landscapes from the perspective of the nucleosome and find an underlying positive correlation between methylated CpG density and nucleosome occupancy. We also acknowledge exceptions to this pattern that can be linked to the presence or absence of other epigenetic factors. Finally, we extensively characterize the chromatin in CpG islands and at conserved transcription factor binding sites to reveal regulation of DNA methylation and nucleosome occupancy in the vicinities of these genomic landmarks.

## Results

### DNA methylation and nucleosome occupancy at the genome level

A common strategy used to study chromatin from genome-wide high-throughput sequencing data involves designating boundary elements and then characterizing the markers surrounding these sites [27]. An example of this approach is given in Additional file 1: Figure S1, which displays average DNA methylation and nucleosome occupancy levels amid computationally predicted sites for the transcription factor, CTCF. These CTCF recognition sequences often mark the boundaries of topologically associated domains [28]. The results show that these presumptive CTCF sites are flanked by a series of regularly spaced nucleosomes and that methylation at CpG dinucleotides occurs preferentially in the linker regions of these phased nucleosomes [29–31]. The elucidation of these patterns raises the question of what can constitute a chromatin boundary.

**Fig. 1:**
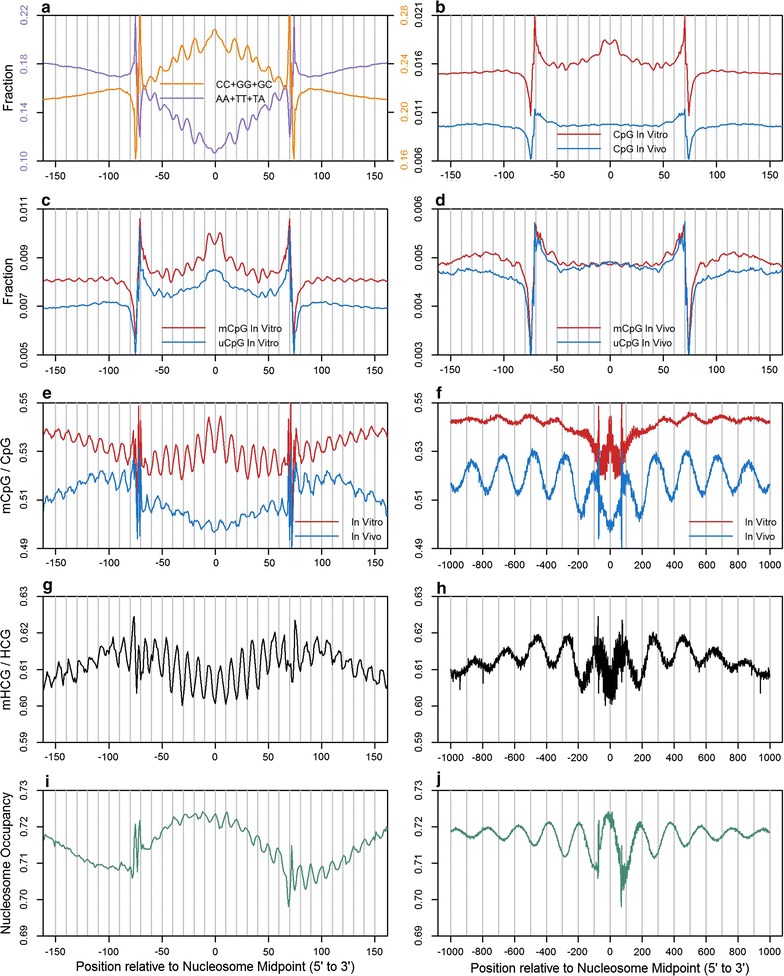
Genome-wide nucleosomal DNA sequence and methylation patterns in leukocytes and IMR90 cells. **a** For the in vitro MNase-seq data from leukocytes, average occurrences of select dinucleotides were computed from forward and reverse complement sequences aligned to nucleosome midpoints. Using BS-seq and MNase-seq data from leukocytes, average occurrences of CpGs (**b**), methylated and unmethylated CpGs (**c**, **d**) and mCpG/CpG fractions (**e**, **f**) were computed for both the in vitro and in vivo libraries from forward and reverse complement sequences aligned to nucleosome midpoints. Using MNase-seq and NOMe-seq data from IMR90 cells, average mHCG/HCG fractions (**g**, **h**) and uGCH/GCH fractions (**i**, **j**) were computed from forward and reverse complement sequences aligned to MNase-seq derived nucleosome midpoints. Nucleosomes within CpG Islands were excluded from this analysis

Approximately 15 million nucleosomes are positioned along the haploid human genome. The primary bioinformatics approach utilized in this work treated each nucleosome identified by MNase-seq as a chromatin boundary element. Using the flanking DNA centered on nucleosome midpoints, the sequence content, DNA methylation levels, and nucleosome occupancies were measured for the entire genome and for several subgenomic regions. In order to compare DNA methylation patterns derived from nucleosomes reconstituted in vitro to those formed in the cell, BS-seq [32] and MNase-seq [33], data derived from leukocytes were used (Fig. 1a–f). For the analysis of nucleosome occupancy and DNA methylation, in vivo MNase-seq and NOMe-seq data from fetal lung fibroblasts (IMR90 cells) were used (Fig. 1g–j) [29].

A hallmark of rotationally positioned nucleosomal DNA is the ∼10-base-pair periodicity of A/T-rich sequences with minor grooves facing toward the histone octamer alternating with G/C-rich sequences whose minor grooves face away [34–36]. This arrangement is illustrated using the in vitro data presented in Fig. 1a. It was suggested long ago that this pattern facilitates the winding of DNA around the histone octamer. According to this widely accepted model, the minor grooves of CpG dinucleotides should face outwards at positions ±10, ±20, ±30, ±40, ±50, ±60, and ±70 base pairs from the nucleosome midpoint, and this pattern has been observed in *S. cerevisiae* and *C. elegans* which lack CpG methylation [37–39]. To our knowledge, such a clear pattern for *total* CpG occurrence has never been observed in mammalian nucleosomal DNA, and the lack of this periodic pattern is illustrated by the in vitro and in vivo datasets in Fig. 1b. However, a weak ~10-bp periodicity of the methylated CpG fraction is detected in the in vitro data (Fig. 1c), which becomes magnified when the fraction of CpGs that are methylated are quantified in Fig. 1e. This periodicity is indicative of an unusual rotational orientation as the minor grooves of the methylated CpG dinucleotides face the histone surface, in agreement with previous reports in Arabidopsis and human DNA sequences [21, 22, 40]. Note that the frequency of CpGs is ~1.6-fold higher in the in vitro data as compared to the in vivo data (Fig. 1b). Also note that nucleosomes from CpG islands were excluded from the analysis for Fig. 1 and for all other analyses unless otherwise specified.

**Fig. 2:**
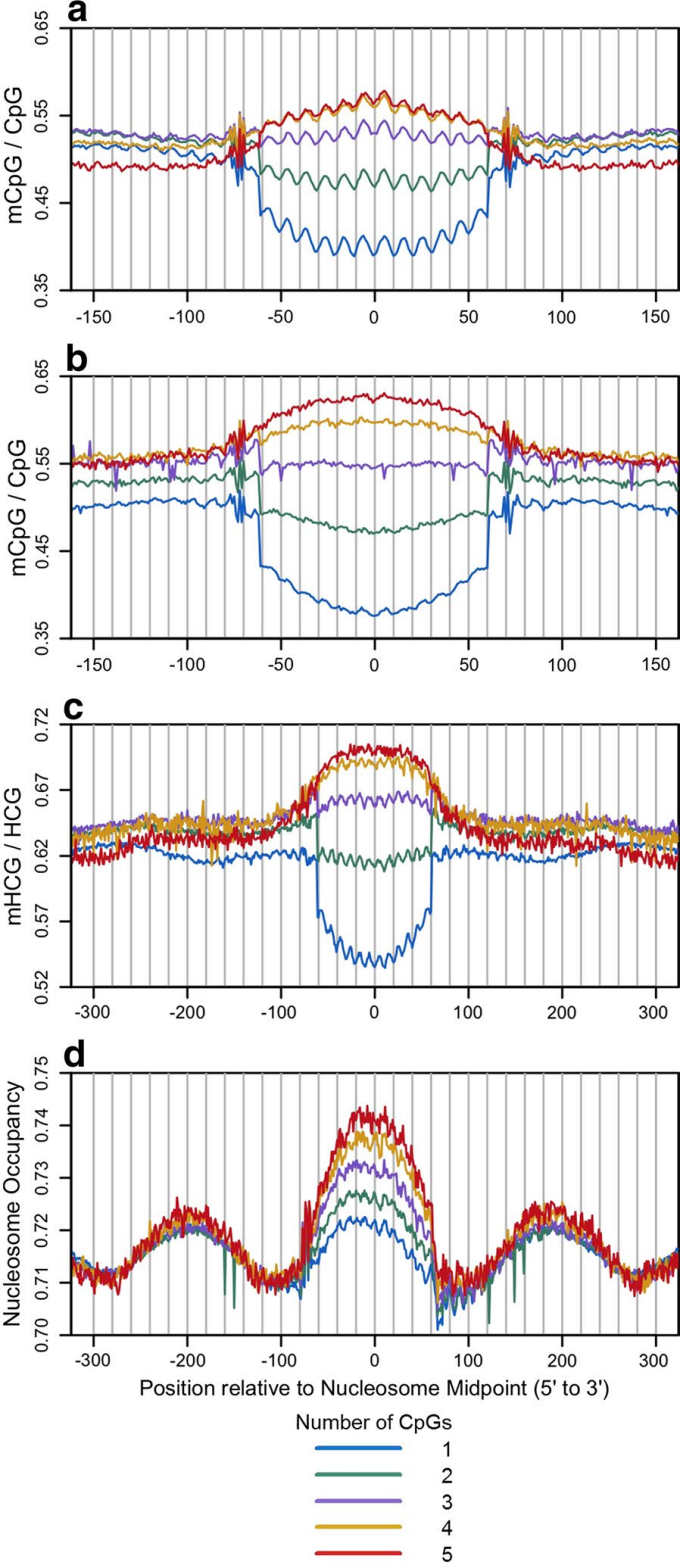
Average DNA methylation levels and nucleosome occupancy as a function of nucleosome core CpG frequency. Nucleosome sublibraries were generated for the in vitro and in vivo leukocyte and IMR90 MNase-seq libraries based on the number of CpG occurrences between positions −61 to +61 relative to nucleosome midpoints. Using BS-seq and MNase-seq data from leukocytes, average mCpG/CpG fractions were computed from forward and reverse complement sequences aligned to nucleosome midpoints for each in vitro (**a**) and in vivo (**b**) nucleosome sublibrary. Using MNase-seq and NOMe-seq data from IMR90 cells, average mHCG/HCG fractions (**c**) and uGCH/GCH fractions (**d**) were computed from forward and reverse complement sequences aligned to MNase-seq derived nucleosome midpoints for each nucleosome sublibrary. Nucleosomes within CpG Islands were excluded from this analysis

**Fig. 3:**
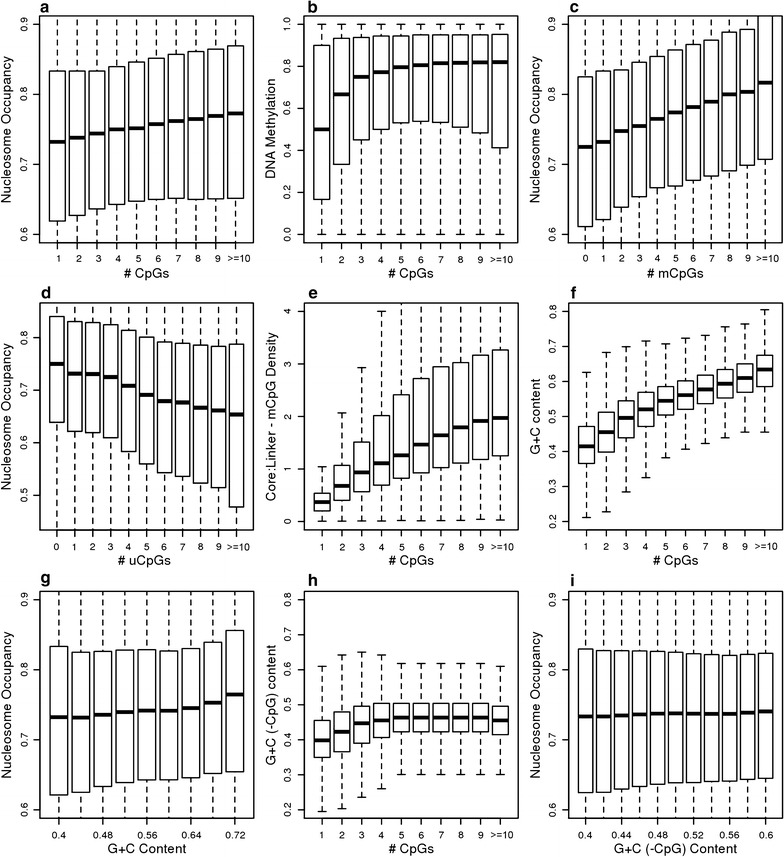
Effects of increasing methylated CpG density in the nucleosome core on nucleosome occupancy and the ratio of methylated CpG density in core versus linker DNA. *Boxplots* display the distribution of nucleosome occupancy as a function of the number of CpGs (**a**), the number of methylated CpGs (**c**), the number of unmethylated CpGs (**d**), G + C content (**g**), and G + C-CpG content (**i**) for all nucleosomes in the genome outside of CpG islands.* Boxplots* also show the distribution of DNA methylation levels (**b**), G + C content (**f**), and G + C-CpG content (**h**) as a function of the number of CpGs. **e** *Boxplots* show the distribution of the ratios of methylated CpG density per base pair in the core versus linker DNA as a function of the number of CpGs. See “Methods” section for more details

Comparisons between the in vitro and in vivo datasets yield striking differences in nucleosomal DNA methylation patterns. Preferential methylation is observed in nucleosome cores from the in vitro dataset (Fig. 1c, e), while a linker preference is found in the in vivo dataset (Fig. 1d, e). This linker preference is extended to nucleosomes that flank the fixed nucleosomes as DNA methylation levels peak between phased nucleosomes (Fig. 1f). On the other hand, nucleosome phasing is not observed in the in vitro data, presumably because the in vitro reconstitutions were carried out in moderate DNA excess. This condition may also explain the enrichment of mCpGs in the nucleosome core from the in vitro dataset since, as shown in Figs. 2 and 3, CpG occurrence is positively correlated with DNA methylation levels and nucleosome occupancy, and as noted earlier, the in vitro data are enriched in CpGs relative to the in vivo data (Fig. 1b). The NOMe-seq data aligned to MNase-seq derived nucleosome midpoints (Fig. 1g–j) mirror the results for the combined BS-seq and MNase-seq data (Fig. 1e, f). Additionally, the nucleosome phasing (Fig. 1j), the enrichment of DNA methylation in linker DNA (Fig. 1h), and the 10-bp periodic patterns of DNA methylation levels with the unique rotational orientation (Fig. 1g) give us confidence that the integration of MNase-seq and NOMe-seq data was precisely executed.

In an attempt to understand the relationship between DNA methylation and nucleosome occupancy, we first divided the MNase-seq derived nucleosome sequences into sublibraries based on the numbers of CpGs. The average mCpG/CpG fractions in the in vitro and in vivo sublibraries from leukocytes were determined, and the results are plotted in panels a and b of Fig. 2, respectively. In both cases, low CpG densities (1–3 CpGs per fragment) correspond to preferred linker methylation, while higher frequencies correspond to preferential methylation in the nucleosome core. These results can explain the preferential linker methylation in the total in vivo data and the preferential core methylation in the total in vitro data since the overall CpG content in the unfractionated in vitro library is 1.6-fold higher than what is observed in the in vivo data (Fig. 1b, e, f). Figure 2c shows essentially the same results using the MNase-seq derived sublibraries and NOMe-seq data from IMR90 cells, and Fig. 2d shows a corresponding graded increase in nucleosome occupancy as a function of increasing CpG content in the central selected nucleosome, and this increase does not appreciably extend into the two flanking nucleosomes.

To further characterize the relationship between DNA methylation and nucleosome occupancy, boxplots were used to show the distributions of nucleosome occupancy in different sequence contexts using all nucleosomes in the genome excluding those within CpG islands (Fig. 3). See “Methods” section for more details. Figure 3a shows an apparent linear relationship between CpG frequency and nucleosome occupancy, while a cooperative relationship between CpG frequency and DNA methylation is indicated in Fig. 3b [20, 39]. Combining mCpG frequency and nucleosome occupancy data in Fig. 3c yields a stronger correlation compared to total CpG frequency (Fig. 3a), suggesting that the presence of mCpGs has a role in dictating nucleosome occupancy levels. This interpretation is further supported by the negative relationship between uCpGs and occupancy (Fig. 3d). An additional feature that is dependent on CpG content is the ratio of mCpG levels in the core versus linker, such that linker preference gradually gives rise to core preference as a function of increasing CpG content (Fig. 3e).

In order to provide balance for the analyses conducted in Figs. 2 and 3, we also investigated the effects of CpG density in linker DNA on nucleosome occupancy and on DNA methylation. Interestingly, with increasing linker CpG density, average nucleosome occupancies remained constant while DNA methylation percentages, for the most part, appeared to drop in the core (Additional file 1: Figure S2). Moreover, when comparing the effects of increasing CpG density in linker versus nucleosome core DNA on mCpG density and on nucleosome occupancy side by side, it becomes apparent that mCpG density in nucleosomal DNA but not linker DNA is correlated with nucleosome occupancy (Additional file 1: Figure S3).

Many studies have implicated G + C content in the control of nucleosome occupancy [41, 42]. However, these models have shown limited applicability in mammalian cells [32]. It is conceivable that the increase in nucleosome occupancy that we observe upon increasing CpG is actually due to an increase in G + C content since CpG-rich fragments tend to be rich in G + C content as computed in Fig. 3f. However, this increase in G + C content is accompanied by only a marginal increase in nucleosome occupancy (Fig. 3g), and when CpGs are removed from the G + C content, there is essentially no considerable effect on nucleosome occupancy as shown in Fig. 3h, i.

### DNA methylation and nucleosome occupancy within various genomic features

**Fig. 4:**
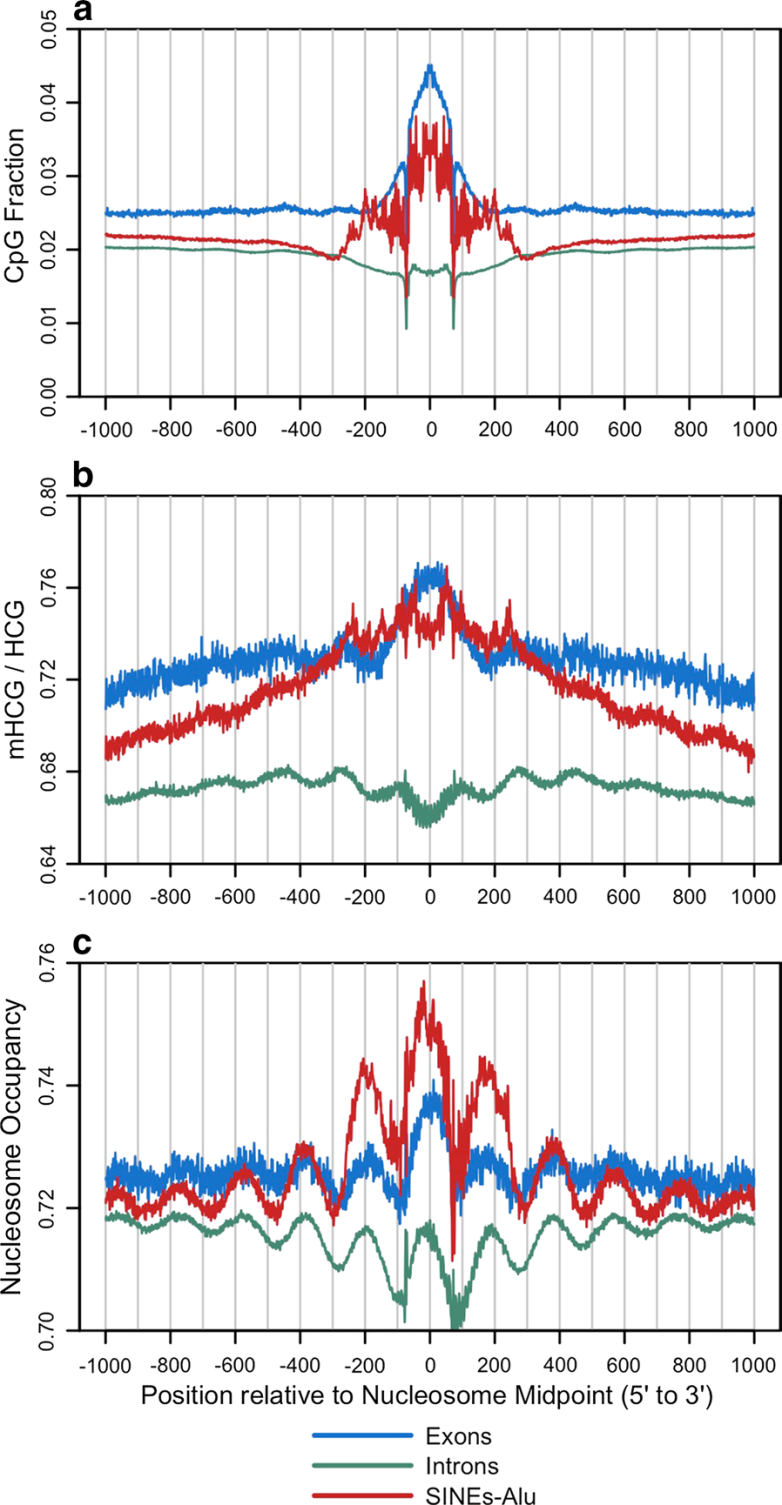
Frequency profiles of CpGs, DNA methylation levels, and nucleosome occupancy surrounding nucleosomes positioned within exons, introns, and SINE-Alu elements. Using MNase-seq and NOMe-seq data from IMR90 cells, average occurrences of CpGs (**a**), mHCG/HCG fractions (**b**) and uGCH/GCH fractions (**c**) were computed from forward and reverse complement sequences aligned to MNase-seq derived nucleosome midpoints

The MNase-seq derived nucleosome midpoints from IMR90 cells were annotated by HOMER [43] in order to determine whether the results for the entire genome displayed in Figs. 2 and 3 are observed within 20 different genomic features (Fig. 4; Additional file 1: Figures S4–S11). We first carried out each of the analyses portrayed by Fig. 3a–i using exons and featureless intergenic sequences in place of the sequences used from the total genome, and the results are similar to those presented in Fig. 3 (Additional file 1: Figures S4, S5). Average profiles of CpG occurrence, DNA methylation, and nucleosome occupancy aligned to nucleosomes within these features reveal a diverse set of chromatin landscapes (Additional file 1: Figures S6–S11), but within each of these genomic features, positive correlations are observed when plotting the distributions of nucleosome occupancy as function mCpG frequency as exhibited in Additional file 1: Figure S12. These results provide additional evidence for the dependence of nucleosome occupancy on mCpG density and imply that this phenomenon is a universal or a nearly universal property in the genome.

Among the genomic features with the highest nucleosome occupancies and mCpG density include exons [22–24] and SINE-Alu transposable elements [44]. Characteristics of these elements along with intron sequences are shown in Fig. 4. As compared to flanking and bulk sequences, both exons and SINE-Alu elements are enriched in CpGs, possess high levels of DNA methylation within the nucleosome core, and display an enrichment in nucleosome occupancy. In contrast, intron sequences are similar to bulk DNA, possessing low CpG content, average nucleosome occupancy, and higher methylation levels in linker DNA. The results in Additional file 1: Figure S13 also show that there are sharp increases in CpG occurrence, DNA methylation, and nucleosome occupancy in exons over introns at both the 5′ and 3′ exon–intron junctions. The higher CpG occurrences in exons as compared to introns are at least partially due to coding constraints. The potential consequences of this effect in terms of the control of co-transcriptional splicing by DNA methylation have been discussed previously [24].

### DNA methylation and nucleosome occupancy in domains of selected histone modifications

**Fig. 5:**
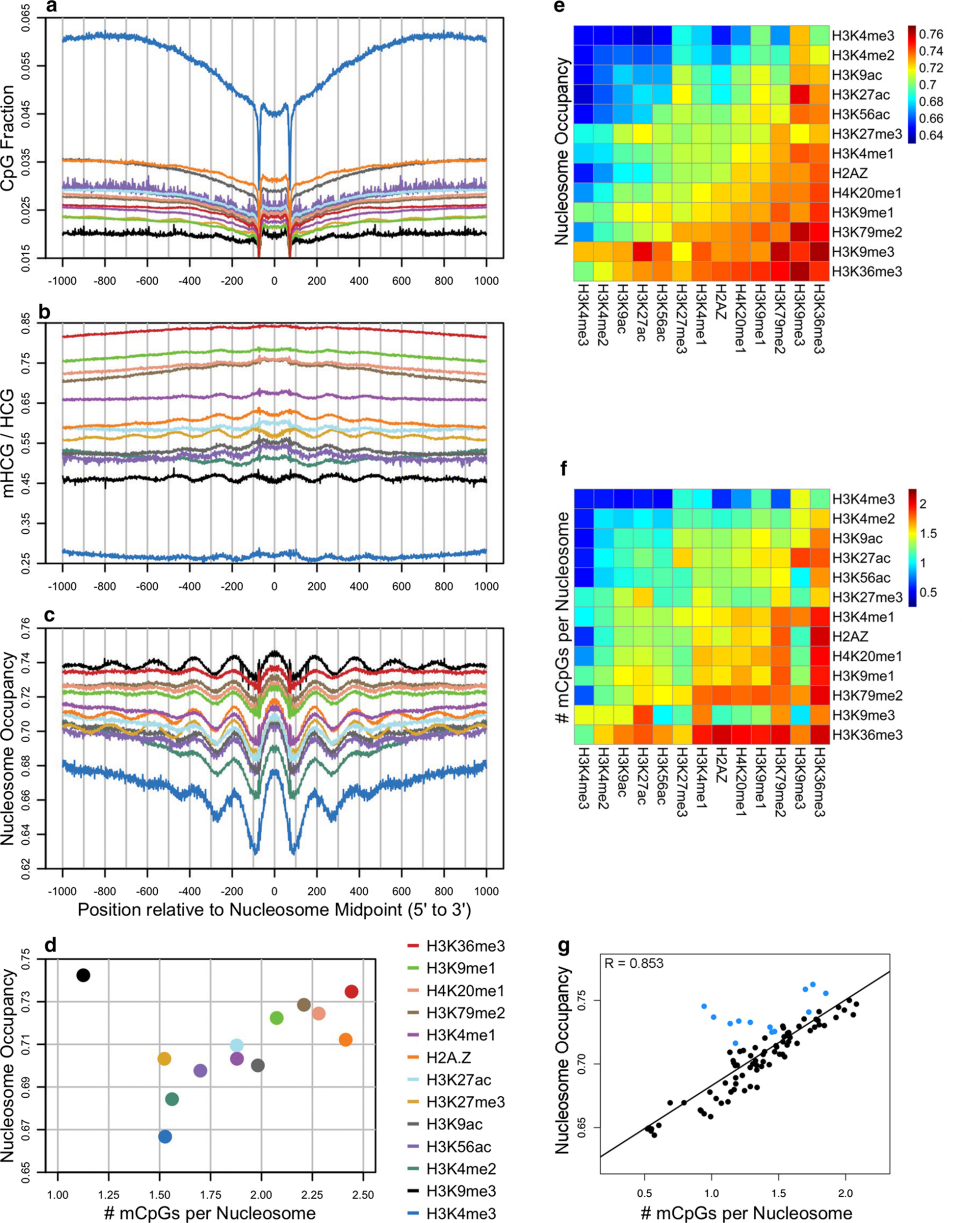
Analysis of nucleosome occupancy and mCpG density in differentially marked chromatin across the genome. Using MNase-seq, NOMe-seq, and Roadmap ChIP-seq data from IMR90 cells, average occurrences of CpGs (**a**), mHCG/HCG fractions (**b**), and uGCH/GCH fractions (**c**) were computed from forward and reverse complement sequences aligned to MNase-seq derived nucleosome midpoints within several histone modification peaks indicated in the key. The 12 histone modifications (and variant H2A.Z) in the key are ordered by decreasing average DNA methylation at the nucleosome midpoint. **d** Using data between positions −61 to +61 relative to the nucleosome midpoint, the average nucleosome occupancy for each histone modification was *plotted* against the corresponding average number of methylated CpGs in the central 123 base pairs of the nucleosome. **e**–**g** Nucleosome occupancy (**e**) and mCpGs density (**f**) were weighted by ChIP-seq *Z*-scores for both single and paired histone modifications (see “Methods” section). **g** *Plots* of nucleosome occupancy and mCpG density from the heatmaps in **e** and **f** show that H3K9me3-modified chromatin (*blue dots*) deviates from the correlation observed in the genome-wide data

Nucleosomes containing posttranslationally modified and variant histones are considered to represent key players in the control of the genome [5, 6, 9, 23, 45]. It was therefore of interest to characterize the DNA methylation status of these nucleosomes in order to determine whether or not their occupancies are proportional to mCpG density as it is in the bulk of the genome. Before performing this analysis, we first annotated every base pair in the peaks of 12 well-studied histone modifications and the variant H2A.Z using IMR90 ChIP-seq data from the Roadmap Epigenomics Project [46]. The annotation data shown in Additional file 1: Figure S14 show where these modifications are enriched or depleted relative to the entire genome. Figure 5a–c displays the CpG content, DNA methylation levels, and nucleosome occupancy data surrounding nucleosome midpoints that are located within peaks of the histone modifications listed in the key of panel d. For each histone modification, the average nucleosome occupancy values were plotted as a function of the number of mCpGs per nucleosome (Fig. 5d).

Nucleosomes marked by H3K4me3 are highly enriched in promoters, 5’UTRs, and CpG Islands (Additional file 1: Figure S12) and as expected contain high levels of unmethylated CpGs and possess low nucleosome occupancy values (Fig. 5). On the other hand, nucleosomes marked by H3K36me3 are located in gene bodies and are enriched by a factor of 2 in exons over introns (Additional file 1: Figure S12). Accordingly, H3K36me3-modified nucleosomes contain moderately high levels of methylated CpGs are associated with high nucleosome occupancy (Fig. 5). Regardless of histone modification, occupancy levels for most nucleosomes are correlated with their mCpG frequencies and DNA methylation levels in this analysis (Fig. 5d; Additional file 1: Figure S15). The only major exception is observed with nucleosomes marked by H3K9me3, which display very low levels of CpGs and low DNA methylation levels yet possess the highest nucleosome occupancy out of all the examined histone modifications (Fig. 5d). H3K9me3-modified nucleosomes are preferentially associated with constitutive heterochromatin where they play critical roles in DNA silencing [47].

In order to further investigate the relationship between DNA methylation and nucleosome occupancy within modified nucleosomes, we expanded the analysis in Fig. 5a–d by assessing the pairwise data among the 12 histone modifications and the variant H2A.Z (Fig. 5e–g). Comparison of the heatmaps displaying nucleosome occupancy (Fig. 5e) and mCpG density (Fig. 5f) as well as DNA methylation levels (Additional file 1: Figure S15) reveals a strong correspondence for most histone modification pairs and is supported by the high correlation given in Fig. 5g. The major exceptions to this correspondence include pairs marked by H3K9me3 indicated by the blue dots in Fig. 5g, which reflect the findings in Fig. 5d.

### DNA methylation and nucleosome occupancy in CpG islands

**Fig. 6:**
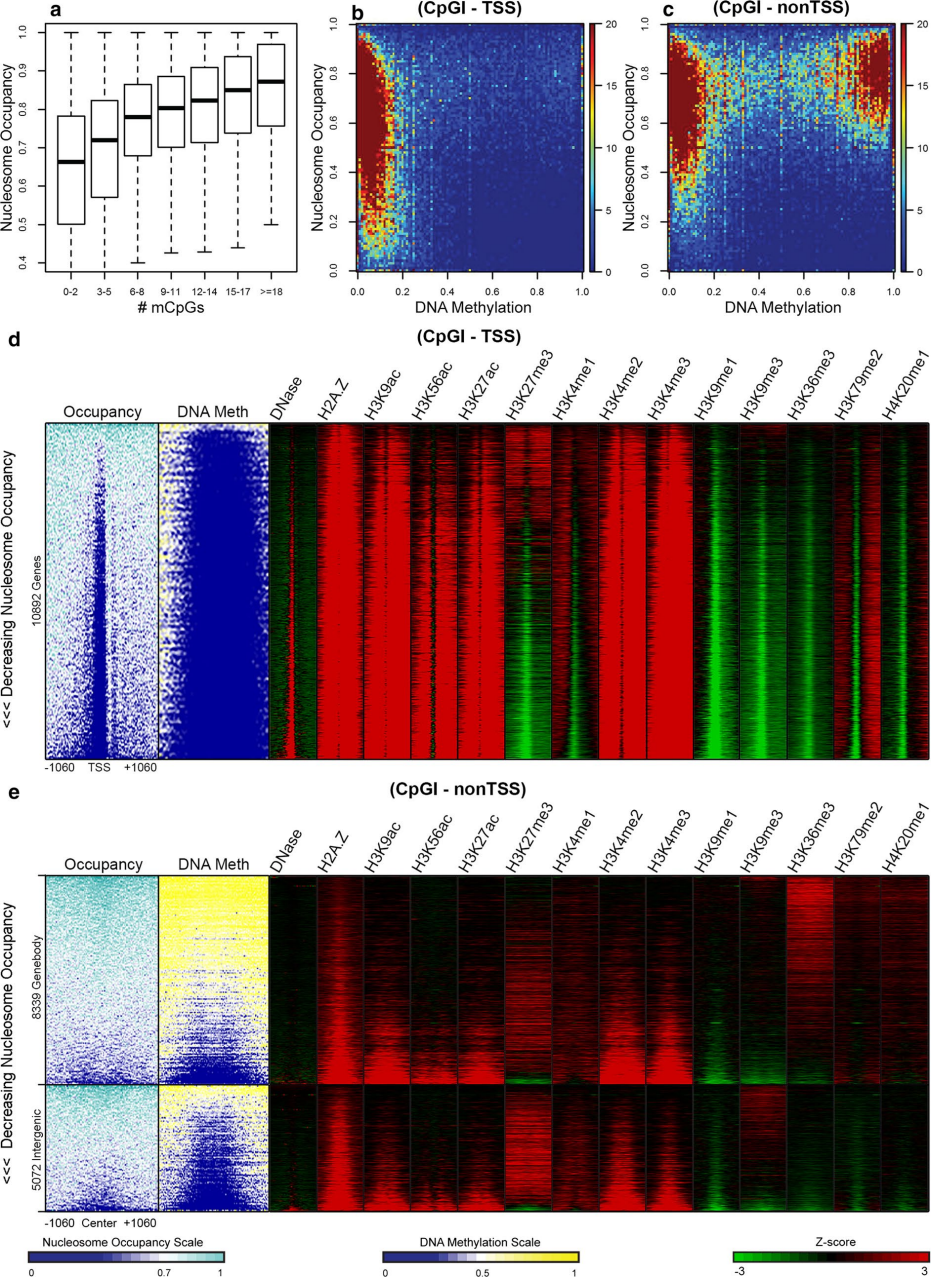
Characterization of chromatin at CpG islands. **a** *Boxplots* display the distribution of nucleosome occupancy as a function of the number of methylated CpGs. **b**, **c** Nucleosome with midpoints within CpG islands was divided into two groups, non-TSS and TSS. For each nucleosome, nucleosome occupancy and DNA methylation values were plotted in the 2D color-coded scatterplots. Using NOMe-seq and Roadmap ChIP-seq data from IMR90 cells, nucleosome occupancy, DNA methylation levels, and *Z*-scores from 12 histone modifications and the histone variant H2A.Z were aligned to TSSs that overlapped CpG Islands (**d**) and to the centers of CpG islands located in gene bodies and intergenic regions (**e**). Heatmap values were computed in 101 21-bp bins surrounding the TSSs and non-TSS CpG island centers, and sites (heatmap rows) were sorted by nucleosome occupancy

Over half of the promoters in the human genome reside within CpG islands. These CpG- and G + C-rich segments serve as platforms for the assembly of unstable nucleosomes and sites for attracting regulatory proteins leading to regions of chromatin that are permissive for transcriptional activation [10]. We characterized the chromatin in CpG islands in IMR90 cells by analyzing the relationships among nucleosome occupancy, DNA methylation, and the various histone modifications analyzed in Fig. 5. Figure 6a shows that, like the bulk of the genome, the frequencies of mCpG are positively correlated with nucleosome occupancies in CpG islands. The CpG island nucleosomes were then divided into two groups, non-TSS and TSS, based on whether or not their midpoints occurred within 500 bp of a TSS. For each MNase-seq derived nucleosome, nucleosome occupancy and DNA methylation values were plotted in 2D color-coded scatterplots (Fig. 6b, c). These results reveal an exception to the theme observed with bulk DNA in that there are significant numbers of the sequences that display low methylation but high nucleosome occupancy.

The heatmaps representing CpG islands displayed in Fig. 6d, e, which consist of NOMe-seq, DNase-seq, and ChIP-seq data sorted by nucleosome occupancy, were constructed in order to elucidate the link between the low DNA methylation levels and high nucleosome occupancies observed in a subset of CpG island nucleosomes (Fig. 6b, c). Figure 6d displays data aligned to TSSs that are overlapped by CpG islands, and Fig. 6e shows data aligned to CpG island centers in non-TSS regions, which were subdivided into intragenic and intergenic groups. An open chromatin configuration at promoters is signified by the void of nucleosomes and the enrichment in DNaseI hypersensitivity at the TSSs in Fig. 6d. The heatmaps appear to show a strong overall correspondence between nucleosome occupancy and DNA methylation in gene bodies and intergenic regions but not at TSSs with high nucleosome occupancy. Most histone modifications appear to follow the overall positive correlation between DNA methylation and nucleosome occupancy. For example, the nucleosomes marked by acetylated histones as well as by H3K4me2/3 are located in unmethylated CpG islands with low nucleosome occupancy, while nucleosomes marked by H3K36me3 are located in methylated CpG islands with moderate to high levels of both occupancy and methylation in gene bodies. One noticeable exception in Fig. 6d is enrichment of H3K27me3, which is an epigenetic mark of polycomb-repressed genes [48]. However, when the heatmaps are sorted by H3K27me3 or H3K9me3 signals, it becomes apparent that both of these epigenetic modifications are characterized by low levels of methylation and moderate to high nucleosome occupancy in CpG islands in all three annotations (Additional file 1: Figures S16, S17) with a stronger anti-correlation associated with the presence of H3K27me3 [49].

### DNA methylation and nucleosome occupancy at conserved transcription factor binding sites

**Fig. 7:**
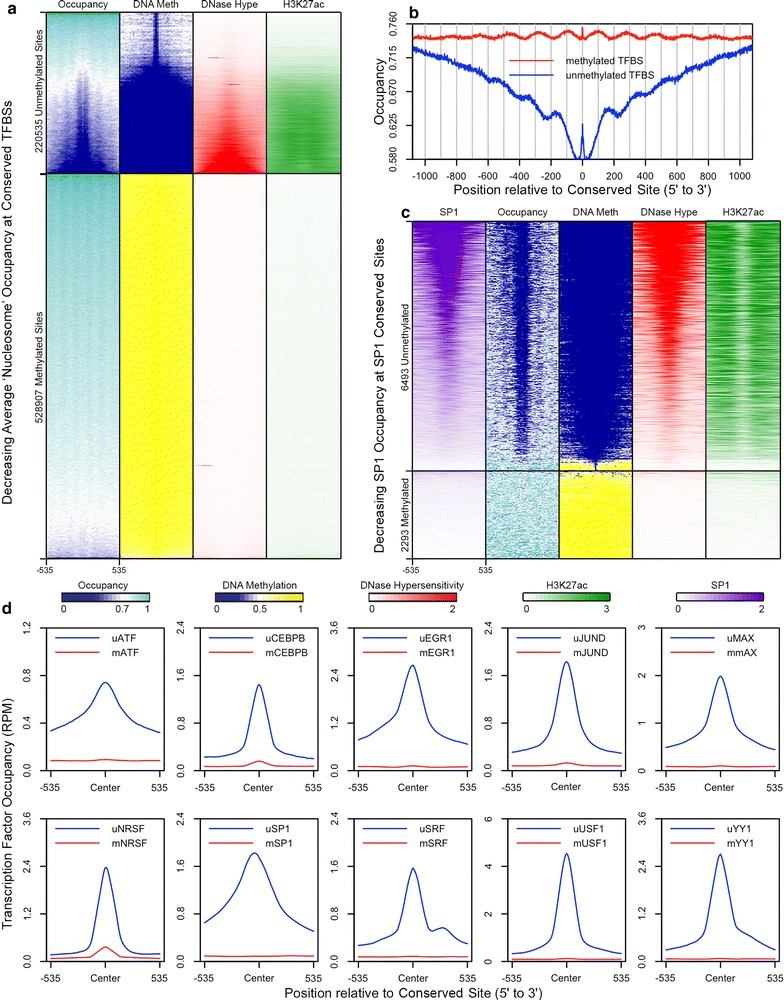
Characterization of chromatin at unmethylated and methylated conserved transcription factor binding sites. All conserved transcription factor binding sites (TFBSs) from the UCSC genome browser were divided into unmethylated and methylated sites (see “Methods” section). **a** Using NOMe-seq, BS-seq, and ENCODE ChIP-seq data from HCT116 cells, nucleosome occupancy, DNA methylation levels, and DNase-seq and H3K27ac signals were aligned to unmethylated and methylated conserved TFBSs. **b** Average uGCH/GCH fractions were also computed from forward and reverse complement sequences aligned to these unmethylated and methylated conserved TFBSs. **c** Similar to **a**, nucleosome occupancy, DNA methylation levels, and DNase-seq, H3K27ac, and SP1 signals were aligned to unmethylated and methylated SP1 conserved TFBSs. **d** Using ENCODE ChIP-seq data for 10 transcription factors, including SP1, average signals were plotted with respect to their corresponding unmethylated and methylated conserved TFBSs. In the heatmaps, average signals were computed in 51 21-bp bins surrounding each conserved site. Scales for DNase-seq, H3K27ac, and the transcription factors represent occupancy levels in reads per million

In order to further evaluate the control of DNA methylation and nucleosome occupancy at regulatory elements in an unbiased manner, we studied all transcription factor binding sites (TFBSs) that are conserved in mammals and that contain CpG in their recognition sequences [50–52]. In this analysis, we characterized the chromatin at TFBSs in HCT116 cells using NOMe-seq [53], BS-seq [54], and also ChIP-seq data from ENCODE (Fig. 7) [55]. All CpG-containing TFBSs provided by the UCSC genome browser were divided into unmethylated and methylated sites (see “Methods” section), and the data above were aligned to these coordinates and sorted by decreasing nucleosome occupancy (Fig. 7a). Nearly all methylated sites display high nucleosome occupancy, while a large fraction of unmethylated sites are nucleosome depleted, DNaseI hypersensitive, and marked by H3K27ac (Fig. 7a, b), in agreement with several studies that have characterized the chromatin at active enhancers [56–58]. We also examined the occupancy levels of 10 transcription factors from ENCODE at their respective unmethylated and methylated CpG-containing TFBSs (Fig. 7c, d). All ten transcription factors exhibit binding at their unmethylated sites and appear nearly or completely absent at their methylated sites (Fig. 7d). Further analysis of the SP1 transcription factor shows that its occupancy is associated with low nucleosome occupancy, unmethylated CpGs, DNaseI hypersensitivity and H3K27 acetylation (Fig. 7c).

We extended the characterization of the chromatin shown in Fig. 7a by examining 5 additional histone modifications and the variant H2A.Z using ChIP-seq data from the Jones laboratory (Additional file 1: Figure S18) [53]. For the methylated sites, the enrichment of H3K36 trimethylation and depletion of H3K27 acetylation and H3K4 trimethylation imply that several methylated sites are located in the gene bodies and deficient in active promoters or enhancers (Additional file 1: Figure S18). On the other hand, the unmethylated sites can be divided into two main groups. One group is enriched in CpG islands as well as active promoters and enhancers, which are indicated by low nucleosome occupancy and DNA methylation with high H3K27 acetylation and H3K4 methylation, and the second group is less CpG rich and possesses low to moderate levels of DNA methylation and high nucleosome occupancy (Additional file 1: Figure S18). Thus, the second group represents another exception to the positive correlation between methylated CpGs and nucleosome occupancy. Interestingly, the second group exhibits an enrichment of H3K9 and H3K27 methylation (Additional file 1: Figure S18), which are the same modifications linked to the exceptions observed in CpG-poor genomic regions and in CpG islands (Figs. 5, 6). These modifications along with the presence of H3K4me1 suggest that some of the sites in the second group may signify poised enhancers (Additional file 1: Figure S18) [56–58].

## Discussion

### Relationships between DNA methylation and nucleosome occupancy in vitro and in vivo

In order to explore links between DNA methylation and nucleosome occupancy, we relied heavily on data derived from NOMe-seq because this methodology, developed by Jones and coworkers, enables the simultaneous measurement of nucleosome occupancy and endogenous methylation for cell populations and single cells [29, 53, 59]. In comparison with NOMe-seq, MNase-seq requires higher coverage and relies on enrichment-based measurements of nucleosome occupancy, and these estimations can be skewed by sequence biases generated by enzyme cutting preferences, extents of digestion, and library amplification steps [60]. However, MNase-seq’s primary advantage comes from its capability to determine the positions of nucleosome midpoints at near base-pair resolution, and this precision can be enhanced if paired-end sequencing is applied [21, 60]. By exploiting the strengths of both MNase-seq and NOMe-seq, we were able to confidently examine the effects of DNA methylation on nucleosome occupancy (Figs. 1, 2, 3; Additional file 1: Figures S2, S3).

Epigenetic factors must be maintained during development and cell differentiation, for without this characteristic they would be diluted during cell division. Two major epigenetic factors that satisfy this criterion are DNA methylation and stable posttranslationally modified histones. The metabolically stable histone methylation of H3K9 and H3K27, in contrast to histones modified by acetylation or phosphorylation, has half-lives measured in hours or longer and is commonly viewed as memory markers [45, 61–64]. These stable histone modifications along with DNA methylation are also involved in chromatin silencing, which raises the question as to whether or not their silencing mechanisms display similarities. The results of this investigation provide implications for this question, for the modes of action of these factors, and for epigenetics in general.

Our results suggest that the nucleosome serves as an effector arm of epigenetic mechanisms and that enhanced nucleosome occupancy is causally related to silencing induced by DNA methylation and stable histone modifications. The results in our previous studies demonstrated that DNA methylation enhances the stability of nucleosomes in a fraction of the human genome in vitro that contains multiple CpGs arranged in an unusual rotation with their minor grooves facing toward the histone octamer [21]. The studies described in this report elaborated on these features and extended the findings to the entire genome under in vivo conditions. The results show that nucleosome occupancy is correlated with mCpG frequency in the total genome and in subgenomic regions defined by CpG frequency (Figs. 2, 3; Additional file 1: Figures S2, S3) and by the coordinates of annotated features (Figs. 4, 6; Additional file 1: Figures S4–S13, S16, S17, S19), histone modification domains (Fig. 5; Additional file 1: Figure S15), and CpG-containing transcription factor binding sites (Fig. 7; Additional file 1: Figure S18). However, our analyses uncovered two exceptions to the generalization between mCpG levels and nucleosome occupancy, which were nucleosomes marked by H3K9me3 and H3K27me3 (Figs. 5, 6; Additional file 1: Figures S16–S18). These nucleosomes displayed low levels of mCpG but high nucleosome occupancy values, which is indicative of some feature, other than DNA methylation, being responsible for their high nucleosome residency.

### Modes of action for mCpG

The results in this report raise the question of why increasing methylated CpG density is associated with increasing nucleosome occupancy. One possible answer could be derived from the influential silencing mechanisms of methyl-binding proteins [65, 66]. It is also conceivable that highly methylated CpG-rich DNA could directly enhance nucleosome stability before nucleosomes are assembled as demonstrated in vitro [21, 67, 68] or become more stable after de novo methylation. We proposed in our MNase-seq study with in vitro reconstituted nucleosomes [21] that the hydrophobic, bulky methyl groups in the accessible major groove could cause narrowing of the corresponding minor groove. Indeed, in a recent DNase-seq experiment conducted on naked DNA, cutting frequencies were shown to be influenced by the methylation-induced narrowing of the minor groove [69]. The consequence of this action could strengthen the interactions between positive-charged histone arginines and the negative-charged DNA phosphate backbone, thereby enhancing nucleosome stability and in turn, increasing nucleosome occupancy in the cell [21, 69].

Although previous studies have indicated that the nucleosome core is the preferred site of DNA methylation [21, 22, 70, 71], others have suggested that methylation occurs preferentially in the linker regions [30, 72]. The results in Figs. 2 and 3 provide a possible explanation for this apparent discrepancy since a transition of preferential methylation from linker to core as a function of increasing CpG content was revealed along with a corresponding increase in nucleosome occupancy. For example, nucleosomes in CpG-rich exonic DNA and SINE-Alu sequences display higher mCpG density and nucleosome occupancy levels in the core, while nucleosomes in CpG-poor intronic DNA show selective methylation in linker DNA (Fig. 4). In light of this trend, the in vivo data displayed in Fig. 1d imply that methylated CpG dinucleotides have virtually no effect on nucleosome occupancy or positioning at the unfractionated genome level. However, in increasingly CpG-rich DNA, the effect of methylated CpG density on nucleosome occupancy becomes more apparent, and therefore, the majority of this effect is likely limited to a small fraction of the genome where CpG density is high. In fact, only ~7% of the genome is represented by 4 or more CpGs in 123-base-pair sliding windows.

The transition of preferential methylation from linker to nucleosome core may reflect the cooperative binding and enzymatic activities of the de novo DNA methyltransferases DNMT3a and DNMT3b, which have been shown to increase with CpG density outside of CpG islands [20]. Similar to bulk genomic DNA, we find a similar cooperative relationship between DNA methylation levels and CpG frequency in nucleosomal DNA (Fig. 3b). This cooperative mode of binding and methylation may be due to the heteromeric nature of the DNMT3 complexes in which two DNMT active sites display a spacing equivalent to about 10 nucleotides of DNA [73]. Thus, in the domains of high CpG density, clusters of CpGs would tend to become preferentially methylated, conceivably promoting nucleosome assembly or repositioning to the site. The unusual rotational orientation of mCpGs in a 10-nucleotide period reported previously [20, 21] and shown in Fig. 1 may also facilitate the action of the DNMTs since multiple mCpGs with major grooves facing away from the histone surface should be the most assessable to these enzymes.

It is important to emphasize that in methylated CpG dinucleotides, the methyl groups reside within the major groove, and it is likely that the major grooves of some CpGs along nucleosomal DNA are less accessible to the de novo methyltransferases. Indeed, it was proposed that the rotational orientation observed in methylated CpGs in Arabidopsis nucleosomes is a product of major groove accessibility [22]. The results of the de novo methyltransferase studies conducted by the Schubeler group imply that in domains of low CpG density, de novo methyltransferase binding and activity is reduced, and consequently, the DNA methylation levels in these regions are expected to be less efficiently maintained [20]. Moreover, with decreasing CpG density, the methylation of nucleosome core DNA may be more effectively inhibited due to the relatively CpG-rich substrate preference of the de novo methyltransferases and due to a decrease in probability of major groove accessibility to CpG dinucleotides. Accordingly, the DNA flanking CTCF sites (Additional file 1: Figure S1), encompassing partially methylated domains (Additional file 1: Figure S19), and in the bulk of the genome (Fig. 1), where DNA methylation levels are higher in linker DNA, are located in CpG-deficient regions. Furthermore, the positive correlation between mCpG density and nucleosome occupancy, as well as the lack of an effect of increasing linker mCpG density on nucleosome occupancy levels, suggests that methylated CpG dinucleotides in adjacent linker DNA are not significantly influencing nucleosome formation (Figs. 2, 3; Additional file 1: Figures S2–S3).

### Genome silencing by DNA methylation, H3K9Me3 and H3K27Me3

A central question concerning epigenetic mechanisms is the source and nature of the primary signals for epigenetic silencing. The signals must ultimately reside in the DNA sequence, but their nature is poorly understood. The signals for relating nucleosome occupancy to DNA methylation are the patterns of CpG dinucleotides, which are encoded in the sequence, and the factors that dictate the methylation status of CpGs such as nucleosome core versus linker localization and the rotational orientation of the CpGs in the nucleosome core. Furthermore, it is most often assumed that the initial signal for posttranslational modification of histones originates with specific regulatory proteins like transcription factors that recognize specific sequences in order to elicit a chain of events that lead to the final modification.

An alternative view is that simple DNA sequence patterns are directly responsible for the initial recognition process [74, 75]. For example, the clustering of unmethylated CpGs in G + C-rich regions is thought to serve as a signal for recognition of polycomb group complexes, which in turn results in the methylation of lysine 27 on histone H3 and ultimately chromatin silencing [48, 75]. Likewise, A + T-rich oligonucleotide sequences have been proposed to play a role in the recognition of H3K9 methylases leading to heterochromatization and repression [74, 75]. It has also been proposed that abundant nuclear proteins such as the HMG box proteins recognize these sites, and it is interesting to note that HMG proteins preferentially bind to AT duplex sequences of the form (ATATAT)N as compared to (AAAAAA)N in physiological ionic strength and temperature and that this AT-heteropolymeric specificity is shared by nucleosomes that contain H3K9Me3 [74, 76].

### DNA methylation, transcription factors, and enhancer chromatin

Active enhancer chromatin typically contains multiple bound transcription factors, certain histone marks such as H3K27ac, undermethylated DNA and an open structure as evidence by DNaseI hypersensitivity [56–58]. We attempted to simplify our analysis by characterizing evolutionally conserved transcription factor chromatin that contain at least one CpG in the recognition sequence. There exists apparent heterogeneity in the dataset as seen by the presence of H3K9me3, H3K27me3, partially methylated DNA, and deficiencies in H3K27ac (Additional file 1: Figure S18), which are characteristics of poised enhancers, sequences which are inactive but have potential for activation. The results in Fig. 7d show that 10 out of 10 transcription factors were preferentially associated with unmethylated DNA sequences, which raises the question whether this specificity is reflected in the binding of transcription factors to naked DNA. There are numerous examples where methylation blocks the in vitro binding of transcription factors that have CpG in their binding sites [77–79], but there are also cases including Sp1 where methylation is without effect on transcription factor binding [79, 80]. The molecular complexity of enhancer chromatin also makes it difficult to unravel cause and effect relationships. It is conceivable that the undermethylation of enhancer DNA is responsible for the reduced nucleosome occupancy and more open chromatin structure of enhancer sequences which would be consistent with the results presented in this study. However, there are alternative explanations to this proposal including the ability of transcription factors to induce loss of methylation at CpG sites, bound transcription factors excluding DNA methyltransferases, and passive DNA demethylation by DNA replication in the absence of maintenance methylation [80, 81].

### Cancer, DNA methylation, and the nucleosome

A conserved property of cancer cells and tumors is global genomic hypomethylation and the local hypermethylation of some CpG Islands [82–84]. The global hypomethylation of the genome is viewed as a driving force for genomic instability in cancer, which characterizes the disease [83]. The activation of many mobile elements such as the SINE-Alu sequences observed in almost all cancers provides examples of this phenomenon [82, 83]. In fact, it has been suggested that the main carcinogenic effect of global DNA hypomethylation in cancer is mediated by its ability to create genomic instability [84]. The results of this study may be relevant to these observations since a reduction in mCpGs levels by as little as a one CpG per nucleosome results in detectable decreases in nucleosome occupancy. A decrease in the occupancy or stability of such nucleosomes might be expected to have effects on the positions of these nucleosomes as well as the positions of nucleosome arrays adjacent to these nucleosomes, which are prevalent in the genome. Changes in the stabilities and positioning of nucleosomes in a significant fraction of the chromatin, as predicted from the present study, are expected to have profound, inheritable effects on the expression of the genome.

## Conclusions

Previous studies have suggested that DNA methylation directly enhances the stability of nucleosomes in vitro. However, the relationship between DNA methylation and nucleosome occupancy is poorly understood. In this study, we implemented a bioinformatics approach that combines MNase-seq and NOMe-seq data to study links between DNA methylation and nucleosome occupancy throughout the human genome. Using this approach, we demonstrated that increasing mCpG density is correlated with nucleosome occupancy and that in mCpG-rich nucleosomes, methylation levels are greater in the core than in the adjacent linker DNA. These nucleosomal DNA methylation patterns were detected not only in total genomic DNA but also within most subgenomic regions. Prominent exceptions to the positive correlation between mCpG density and nucleosome occupancy included CpG islands marked by H3K27me3 and CpG-poor heterochromatin marked by H3K9me3, and these modifications, along with DNA methylation, characterize the major silencing mechanisms of mammalian chromatin. Thus, the density of methylated CpG dinucleotides may be an important factor in regulating nucleosome occupancy levels the human genome.

## Methods

### Data acquisition

Previously aligned in vivo and in vitro MNase-seq nucleosome and control data from leukocyte cells (neutrophil granulocytes) were acquired from GEO under accession number GSE25133 (GSM678045-63) [33]. Processed ENCODE BS-seq data from leukocyte cells (GM12878) were obtained from EMBL-EBI and are associated with GEO accession number GSE40832 (GSM1002650) [32]. Previously aligned MNase-seq data and processed NOMe-seq data from IMR90 cells were obtained from GEO under accession numbers GSE21823 (GSM543311) and GSE40770 (GSM1001125), respectively [29]. Previously aligned DNase-seq and ChIP-seq data for 12 histone modifications and the histone variant H2A.Z from IMR90 cells were obtained from the UCSD Human Reference Epigenome Mapping Project (Roadmap, GSE16256) [46]. Processed NOMe-seq data (for Fig. 7; Additional file 1: Figure S16) and ChIP-seq data (for Additional file 1: Figure S17) from HCT116 cells were obtained from GEO (GSE58638) [53]. Processed BS-seq data (for Fig. 7; Additional file 1: Figure S16) and previously aligned ChIP-seq and DNase-seq data (for Fig. 7) from HCT116 cells were obtained from GEO (GSE60106, GSM1465024) [54] and the ENCODE project [55], respectively. Exon, CpG island, and conserved TFBS coordinates were obtained from the UCSC genome browser [85] and/or the HOMER software [43], and computationally predicated CTCF sites were obtained from the CTCF Database 2.0 [86].

### Identification and annotation of nucleosome midpoints

Using MNase-seq data from leukocyte and IMR90 cells, nucleosome midpoints were determined by adding or subtracting 73 base pairs from the 5′ end of each read that aligned to plus or minus strands, respectively. For the MNase-seq in vitro and in vivo nucleosome and control data from leukocyte cells, coverage was calculated in 147-base-pair windows across the genome, and fractions of coverage between the control and the nucleosome libraries were computed. Subsequently, the numbers of reads at the nucleosome midpoints were normalized by these fractions of coverage in order to subtract background that could be generated by MNase cutting biases. Regardless of whether or not the MNase control data were subtracted from the nucleosome data, the dinucleotide and DNA methylation frequency profiles in Fig. 1a–f appeared nearly identical to ones where control data were not subtracted (data not shown). Nucleosome midpoints from IMR90 cells were annotated using the HOMER software package, and all frequency profiles surrounding nucleosome midpoints were generated using in-house scripts.

### Data analysis for Fig. 3

Using MNase-seq and NOMe-seq data from IMR90 cells, the number of CpGs, G + C content, average mHCG/HCG fraction, and average uGCH/GCH fraction were computed for each nucleosome outside of CpG islands between positions −61 and +61 relative to the MNase-seq derived nucleosome midpoint. Methylation data for at least 2 cytosines within HCGs and GCHs regardless of strand were required for inclusion of a nucleosome in the analysis. With these data, boxplots were used to display the distributions presented in the figure. The numbers of methylated and unmethylated CpGs were determined by multiplying the average mHCG/HCG and uHCG/HCG fractions, respectfully, by the number of CpGs and rounding to the nearest integer. For the analysis in panel e, the number of CpGs and the average mHCG/HCG ratio were computed for the linker DNA of each included nucleosome between positions (±85 to ±115) relative to the nucleosome midpoint. Methylation data for at least 2 cytosines within HCGs in the linker DNA regardless of strand were required for inclusion of a nucleosome in this analysis. With these data, boxplots show the distribution of the ratios of methylated CpG density per base pair in the core versus linker DNA as a function of the number of CpGs. Methylation CpG density per base pair in a nucleosome and its linker DNA were computed by multiplying the average mHCG/HCG ratio by the number of CpGs in the core and linker separately and dividing by 123 and 62 base pairs, respectfully.

### Identification and annotation of ChIP-seq peaks

Peaks for ChIP-seq data from IMR90 cells were identified using SICER with default parameters [87]. Each base pair in every peak called by SICER for the 12 histone modifications and the histone variant H2A.Z was annotated by the HOMER software.

### Data analysis for Fig. 5

For the analysis represented by panels e–g, the number of CpGs, average mHCG/HCG fraction, and average uGCH/GCH fraction were determined for each nucleosome between positions −61 and +61 relative to each MNase-seq derived nucleosome midpoint. ChIP-seq *Z*-scores for the 12 histone modifications and the variant H2A.Z were also determined at each midpoint. For each histone modification, averages values were weighted by *Z*-scores using the following formula.

$$\bar{x}\left( h \right) = \frac{{\mathop \sum \nolimits_{i}^{n} x_{i} \times z\left( h \right)_{i} }}{{\mathop \sum \nolimits_{i}^{n} z\left( h \right)_{i} }}\; \quad {\text{if}}\; z\left( h \right)_{i} > 0$$

For histone modification pairs, average values were weighted by the modification with the smaller *Z*-score.

$${\text{if}}\; z\left( {h_{A} } \right)_{i} \;{\text{and}}\; z\left( {h_{B} } \right)_{i} > 0$$

$$\bar{x}\left( {h_{A,B} } \right) = \frac{{\mathop \sum \nolimits_{i}^{n} x_{i} \times z\left( {h_{A} } \right)_{i} + \mathop \sum \nolimits_{i}^{n} x_{i} \times z\left( {h_{B} } \right)_{i} }}{{\mathop \sum \nolimits_{i}^{n} z\left( {h_{A} } \right)_{i} + \mathop \sum \nolimits_{i}^{n} z\left( {h_{B} } \right)_{i} }}$$

$$z\left( {h_{A} } \right)_{i} = 0\; \quad {\text{if}}\;z\left( {h_{A} } \right)_{i} > z\left( {h_{B} } \right)_{i}$$

$$z\left( {h_{B} } \right)_{i} = 0\; \quad {\text{if}}\;z\left( {h_{B} } \right)_{i} > z\left( {h_{A} } \right)_{i}$$

### Data analysis for Fig. 6

Using MNase-seq and NOMe-seq data from IMR90 cells, the same procedure described for Fig. 3 was carried out for nucleosomes positioned in CpG islands. CpG island nucleosomes were divided into two groups, non-TSS and TSS, based on whether or not their midpoints occurred within 500 bp of a TSS. These information were used to generate the plots in panels a, b, and c. For panel d, if a CpG island overlapped a RefSeq TSS, the CpG island was included. For panel e, if a CpG island center was annotated as an exon or intron (gene body) or as intergenic by HOMER and if the CpG island was not used in panel d, the CpG island was included. Subsequently, NOMe-seq data in bigwig format were aligned to CpG island TSSs and CpG island gene body and intergenic centers using an unpublished Perl script written by Yaping Liu, and in-house scripts were used to express the occupancy and methylation levels in 101 21-base pair bins. Subsequently, the CpG islands were sorted by decreasing average occupancy across the 101 bins. Using Roadmap ChIP-seq data from IMR90 cells in bed format and another Perl script written by Yaping Liu, RPM values minus input were computed across the genome for each dataset, and these values were transformed into *Z*-scores. These *Z*-scores were then aligned to the TSSs and CpG islands centers sorted by decreasing occupancy and binned in the same way as above. Heatmaps were generated using Java Tree View [88].

### Data analysis for Fig. 7 and Additional file 1: Figure S18

BS-seq data from HCT116 were used instead of NOMe-seq data to evaluate the DNA methylation levels at conserved TFBSs so that more sites could be analyzed. All hg19 conserved TFBSs from the UCSC genome browser with methylation data for a cytosine in at least one CpG were divided into unmethylated and methylated sites depending on whether or not the average mCpG/CpG fraction was greater than or equal to 0.5. These conserved TFBSs do not include CTCF conserved sites. Alignment of the NOMe-seq occupancy data to conserved TFBSs was executed in the same way as described for Fig. 6. For Fig. 7, ENCODE ChIP-seq and DNase-seq data in bam format were aligned to conserved TFBSs using ngs.plot [89], but RPM levels were not transformed into *Z*-scores. For Additional file 1: Figure S18, previously generated ChIP-seq *Z*-scores in bigwig format were aligned to the conserved TFBSs, and these values were expressed in 21-bp bins. All heatmaps were generated using Java Tree View [88].

## Supplementary methods

More detailed bioinformatics procedures and in-house scripts used in this study are provided in Additional file 2.

## Additional files

**Additional file 1.** Additional figures.

**Additional file 2.** Detailed bioinformatics procedures and in-house scripts used in this study are enclosed in the zip file.
