# Supplementary material for: Links between DNA methylation and nucleosome occupancy in the human genome
Source: Epigenetics Chromatin. 2017 Apr 11;10:18. doi: 10.1186/s13072-017-0125-5 (PMC5387343; doi:10.1186/s13072-017-0125-5)
Supplement: Supplementary file 1 — Additional file 1. Additional figures. [file 13072_2017_125_MOESM1_ESM.docx]

**Additional Figures**

**Links between DNA methylation and Nucleosome Occupancy in the human genome**


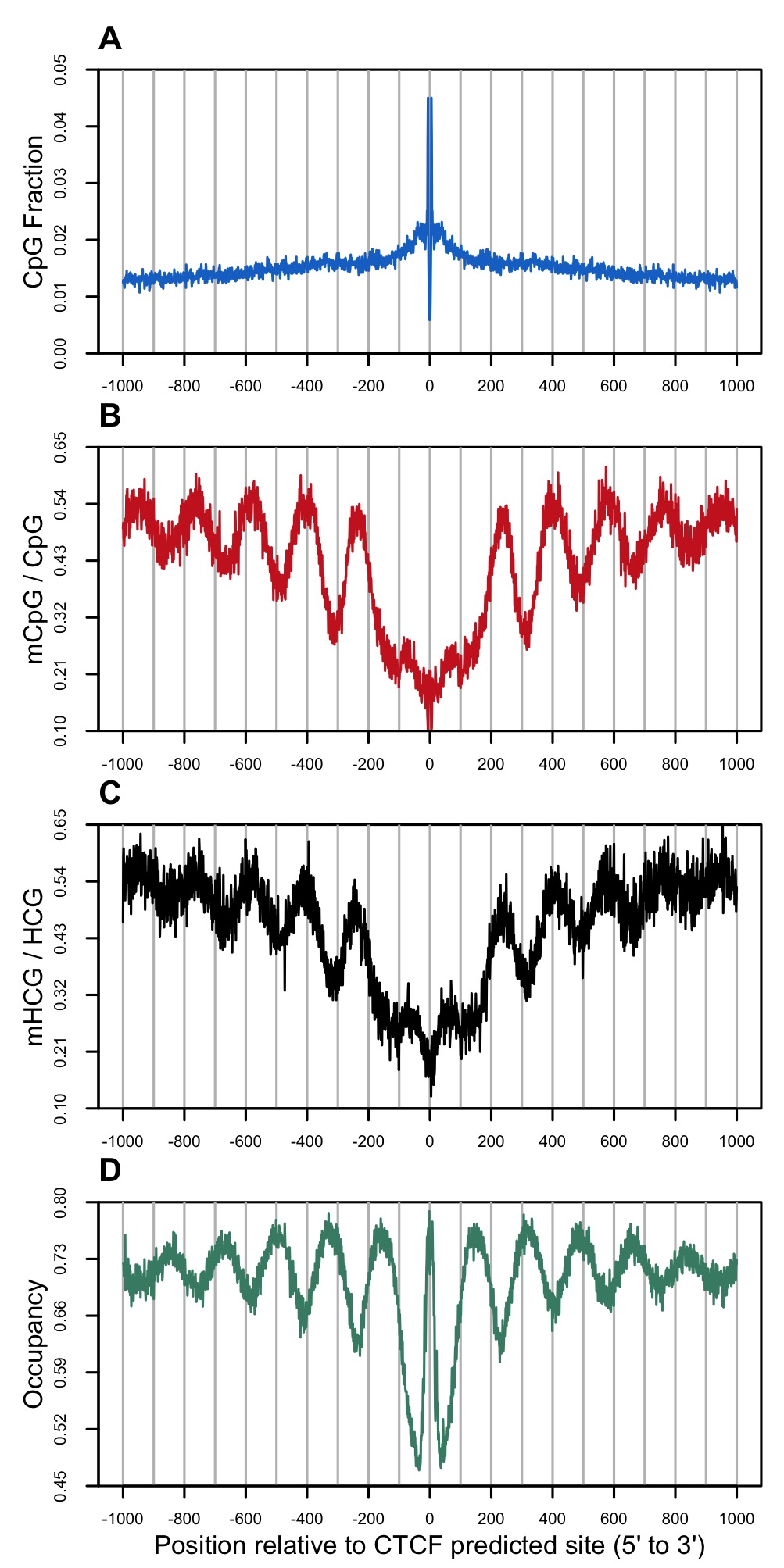


Figure S1. Genome-wide nucleosomal DNA sequence and methylation patterns in GM12878 and IMR90 cells surrounding CTCF sites. A) Average occurrences of CpGs were computed from forward and reverse complement sequences aligned to computationally predicted CTCF sites. B) Using BS-seq and *in vivo* MNase-seq data from GM12878 cells, average mCpG/CpG fractions were computed from forward and reverse complement sequences aligned to computationally predicted CTCF sites. Using NOMe-seq data from IMR90 cells, average mHCG/HCG fractions (C) and uGCH/GCH fractions (D) were computed from forward and reverse complement sequences aligned to computationally predicted CTCF sites.


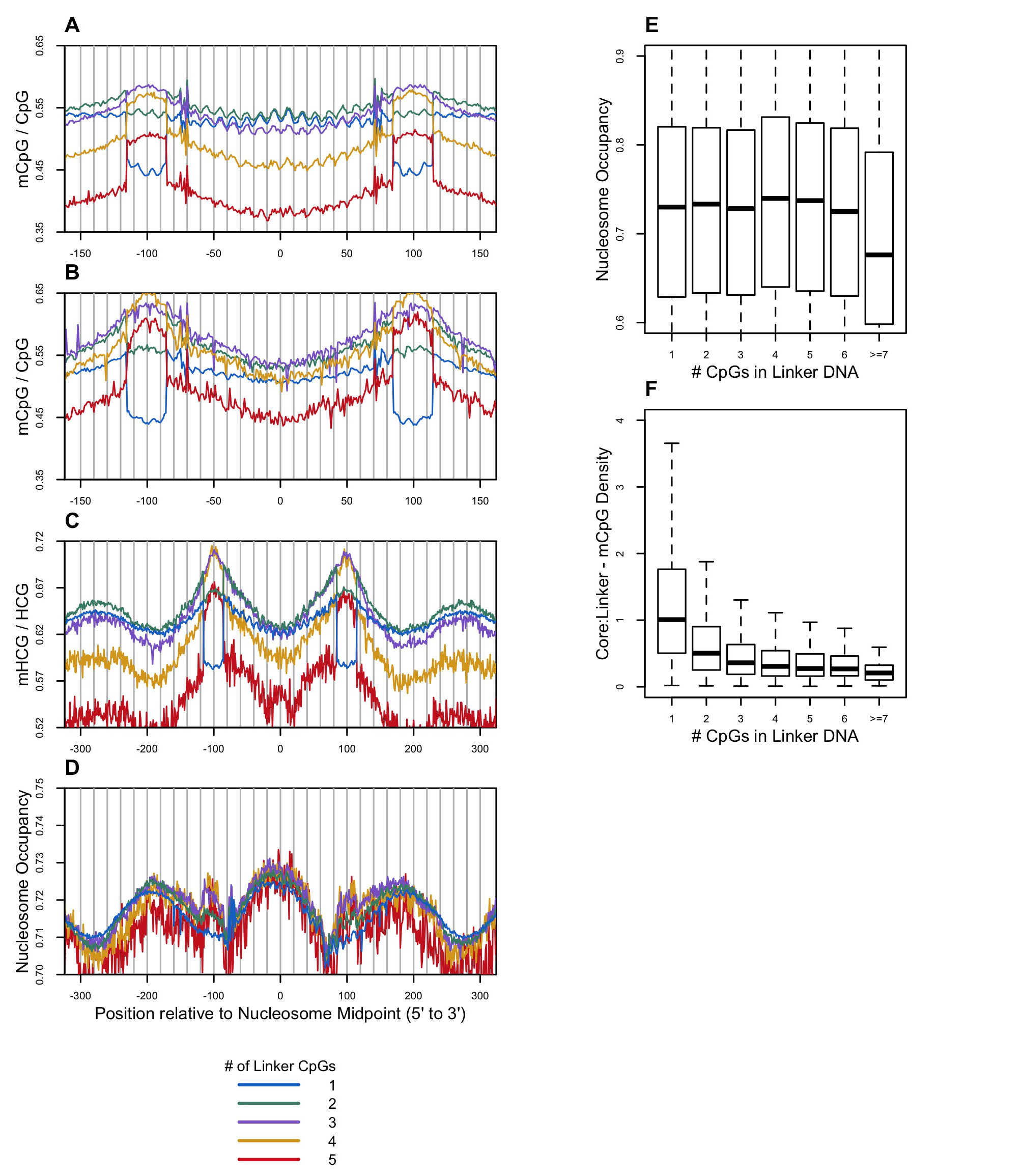


Figure S2. Average DNA methylation levels and nucleosome occupancy as a function of linker CpG frequency. Nucleosome sublibraries were generated for the *in vitro* and *in vivo* leukocyte and IMR90 MNase-seq libraries based on the number of CpG occurrences in linker DNA between positions +/-85 to +/-115 relative to nucleosome midpoints. Using BS-seq and MNase-seq data from leukocytes, average mCpG/CpG fractions were computed from forward and reverse complement sequences aligned to nucleosome midpoints for each *in vitro* (A) and *in vivo* (B) nucleosome sublibrary. Using MNase-seq and NOMe-seq data from IMR90 cells, average mHCG/HCG fractions (C) and uGCH/GCH fractions (D) were computed from forward and reverse complement sequences aligned to MNase-seq derived nucleosome midpoints for each nucleosome sublibrary. Nucleosomes within CpG Islands were excluded from this analysis. E,F) Similar to Figures 3A and 3E, the distribution of nucleosome occupancy levels and core:linker mCpG density ratios were plotted as a function of increasing numbers of CpGs in linker DNA.


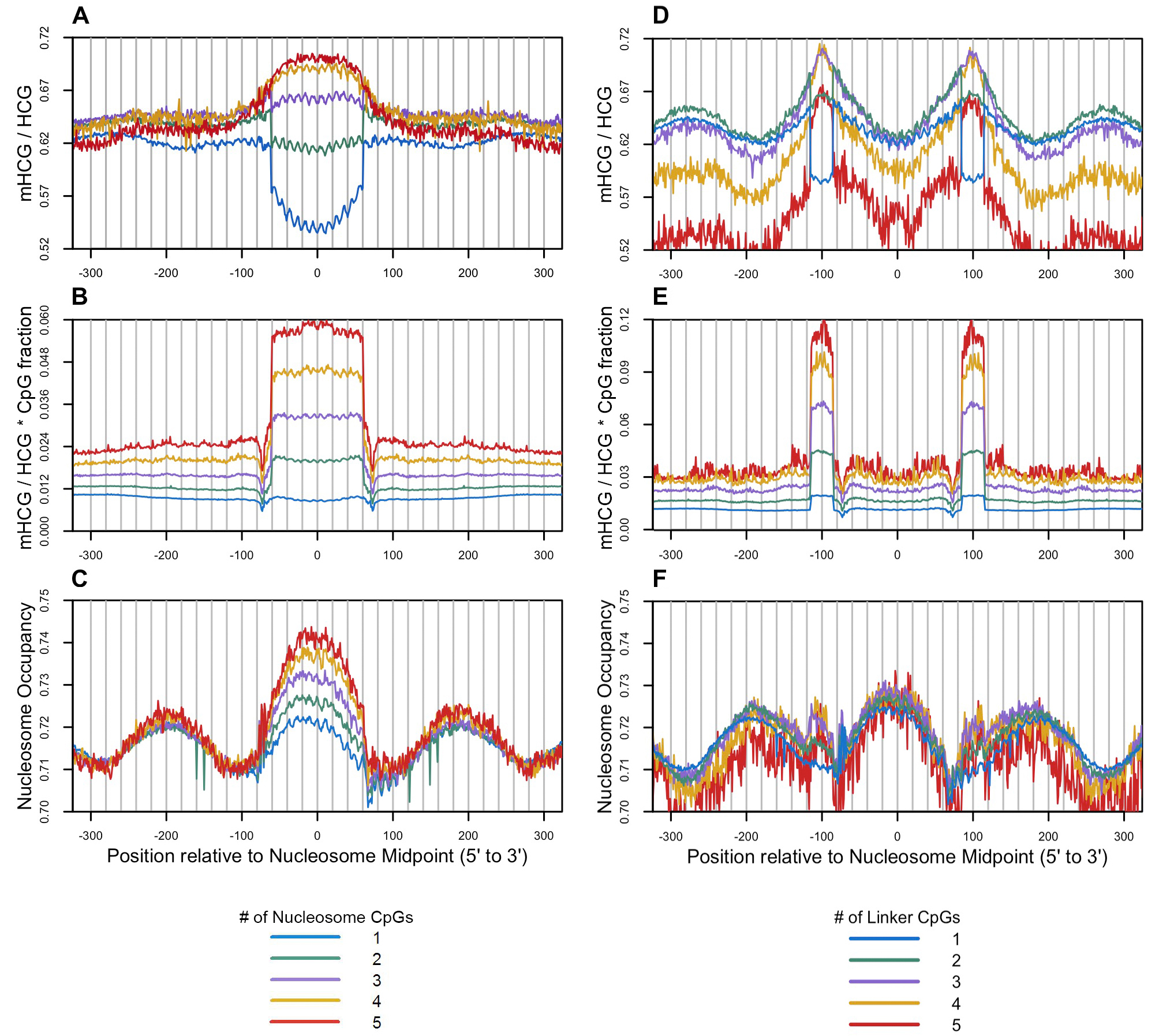


Figure S3. Effects of mCpG density in linker and nucleosome core DNA on nucleosome occupancy. Nucleosome sublibraries were generated for the IMR90 MNase-seq libraries based on the number of CpG occurrences in nucleosomal DNA between positions -61 and +61 relative to nucleosome midpoints (A-C) and in linker DNA between positions +/-85 to +/-115 relative to nucleosome midpoints (D-E). Using MNase-seq and NOMe-seq data from IMR90 cells, average mHCG/HCG fractions (A,D), average mHCG/HCG fractions multiplied by CpG fractions (B,E) and uGCH/GCH fractions (C,F) were computed from forward and reverse complement sequences aligned to MNase-seq derived nucleosome midpoints for each nucleosome sublibrary. Nucleosomes within CpG Islands were excluded from this analysis. Note that due to the constraints of the analysis in Figures 3E and S2F, the methylated CpG density median ratios do not reflect what can be inferred from Figure S3, but the same trend is still observed.


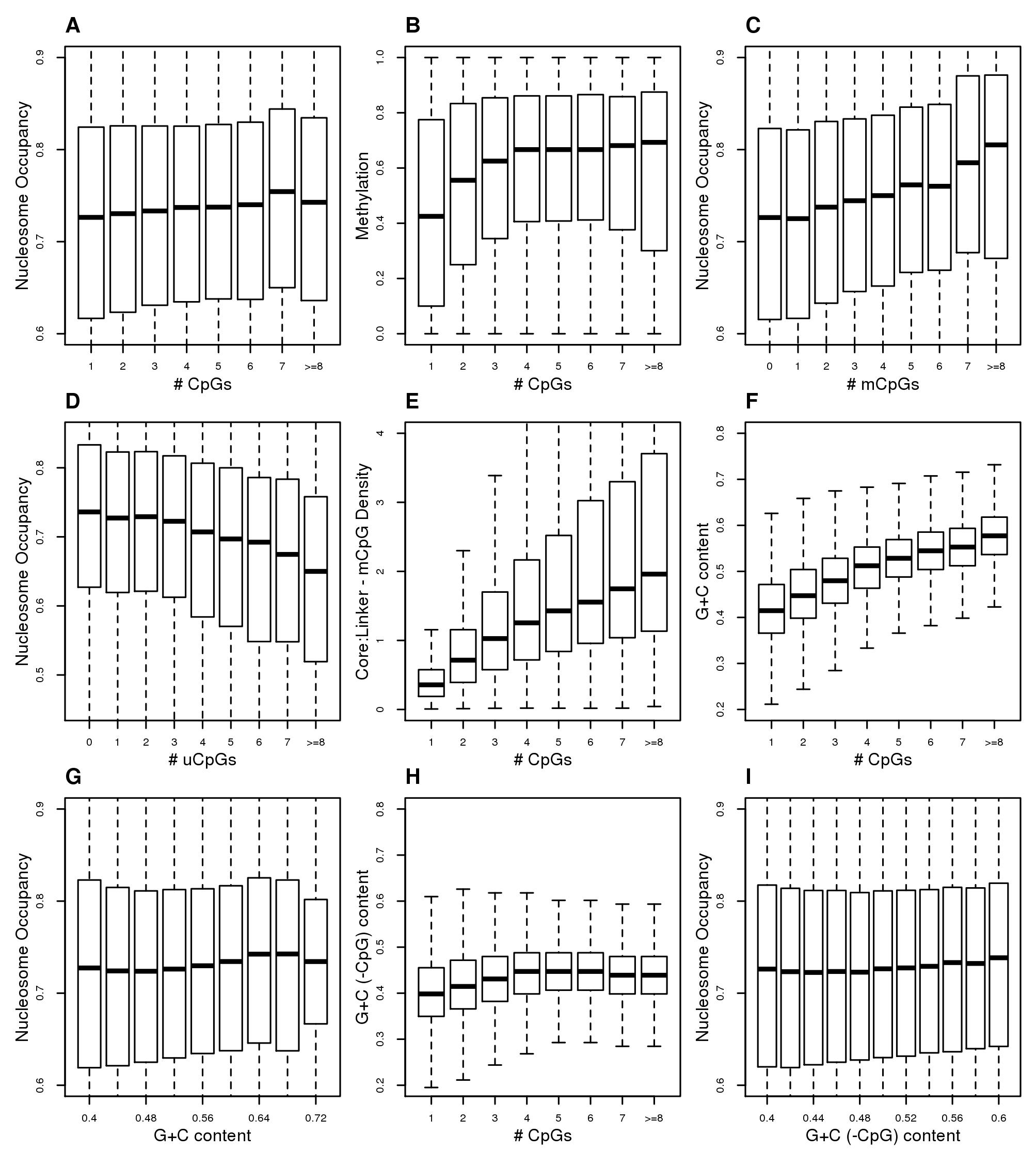


Figure S4. Effects of increasing methylated CpG density in the nucleosome core on nucleosome occupancy and the ratio of methylated CpG density in core versus linker DNA for nucleosomes in intergenic regions. Using MNase-seq and NOMe-seq data from IMR90 cells, the same analysis described for Figure 3 was carried out for nucleosomes positioned in ‘featureless’ intergenic regions as annotated by the HOMER software.


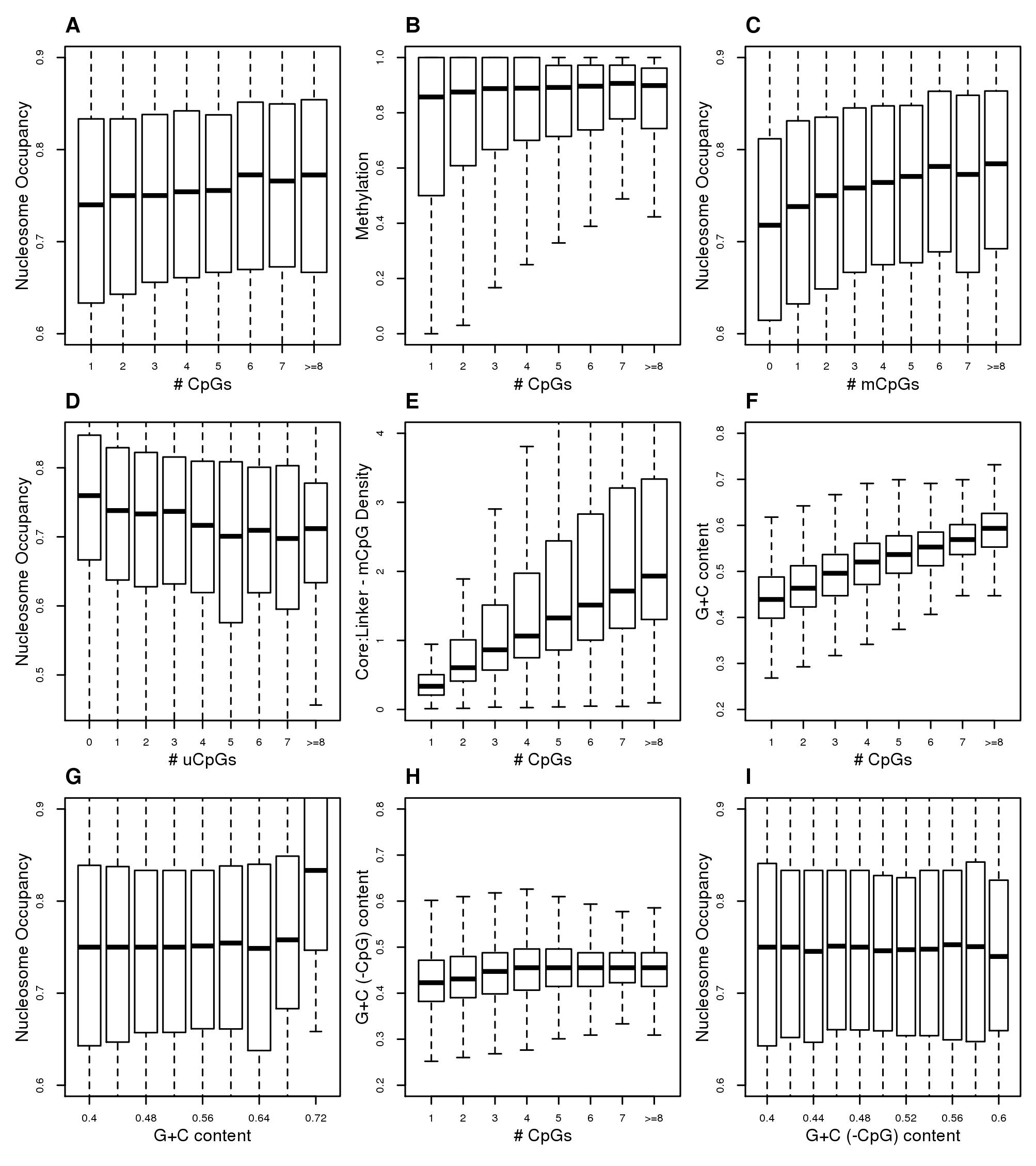


Figure S5. Effects of increasing methylated CpG density in the nucleosome core on nucleosome occupancy and the ratio of methylated CpG density in core versus linker DNA for nucleosomes in exons. Using MNase-seq and NOMe-seq data from IMR90 cells, the same analysis described for Figure 3 was carried out for nucleosomes positioned in exons as annotated by the HOMER software.


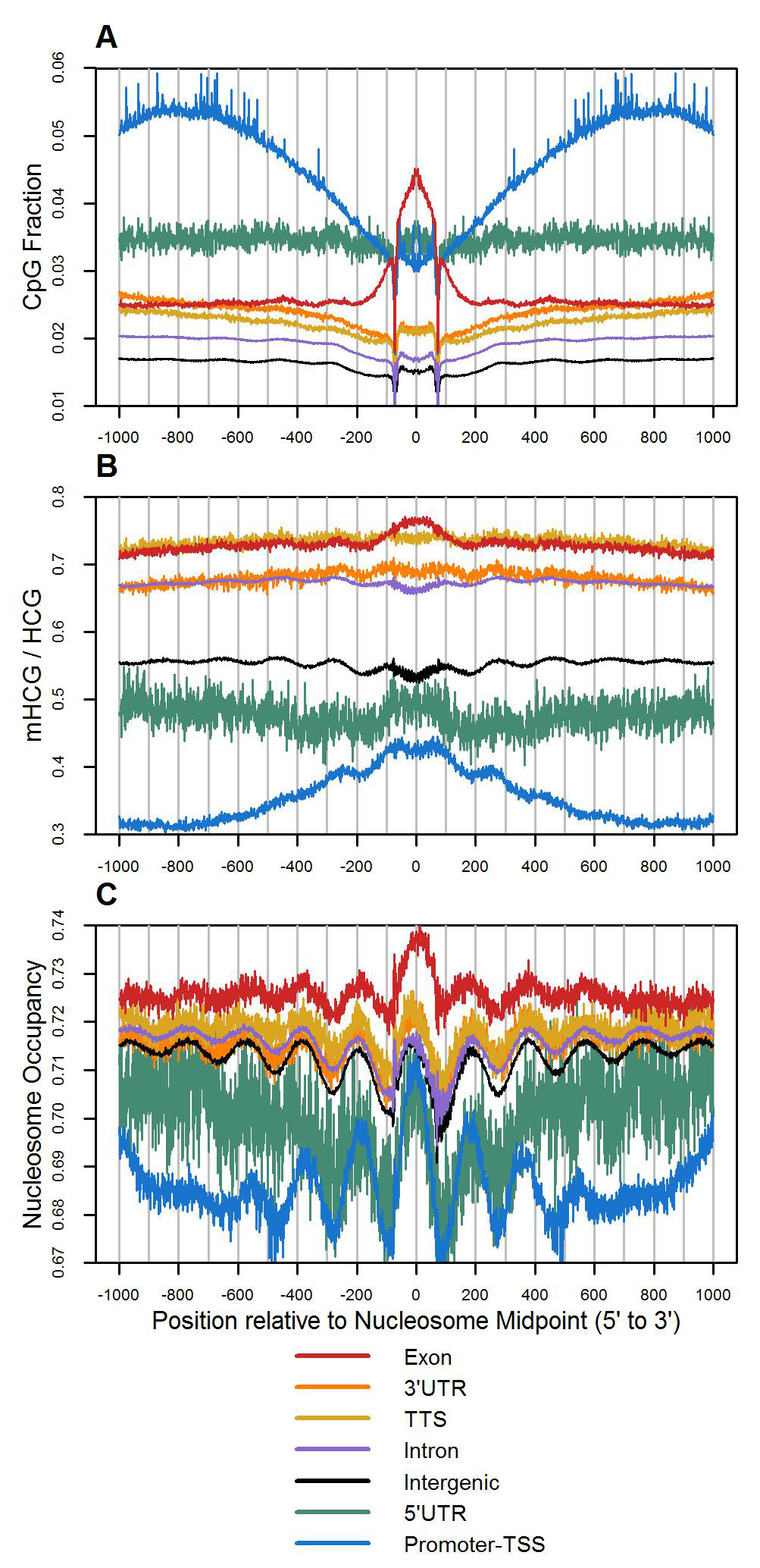


Figure S6. Frequency profiles of CpGs, DNA methylation levels, and nucleosome occupancy surrounding nucleosomes positioned within promoter-TSS, TTS, 5’UTR, 3’UTR, exon, intron, and ‘featureless’ intergenic regions as annotated by the HOMER software. Promoter-TSS regions are defined as -1000 to +100 relative to transcription start sites, and TTS regions are defined as -100 to +1000 relative to transcription termination sites. Using MNase-seq and NOMe-seq data from IMR90 cells, average occurrences of CpGs (A), mHCG/HCG fractions (B) and uGCH/GCH fractions (C) were computed from forward and reverse complement sequences aligned to MNase-seq derived nucleosome midpoints within these features. Nucleosomes within CpG Islands were excluded from this analysis.


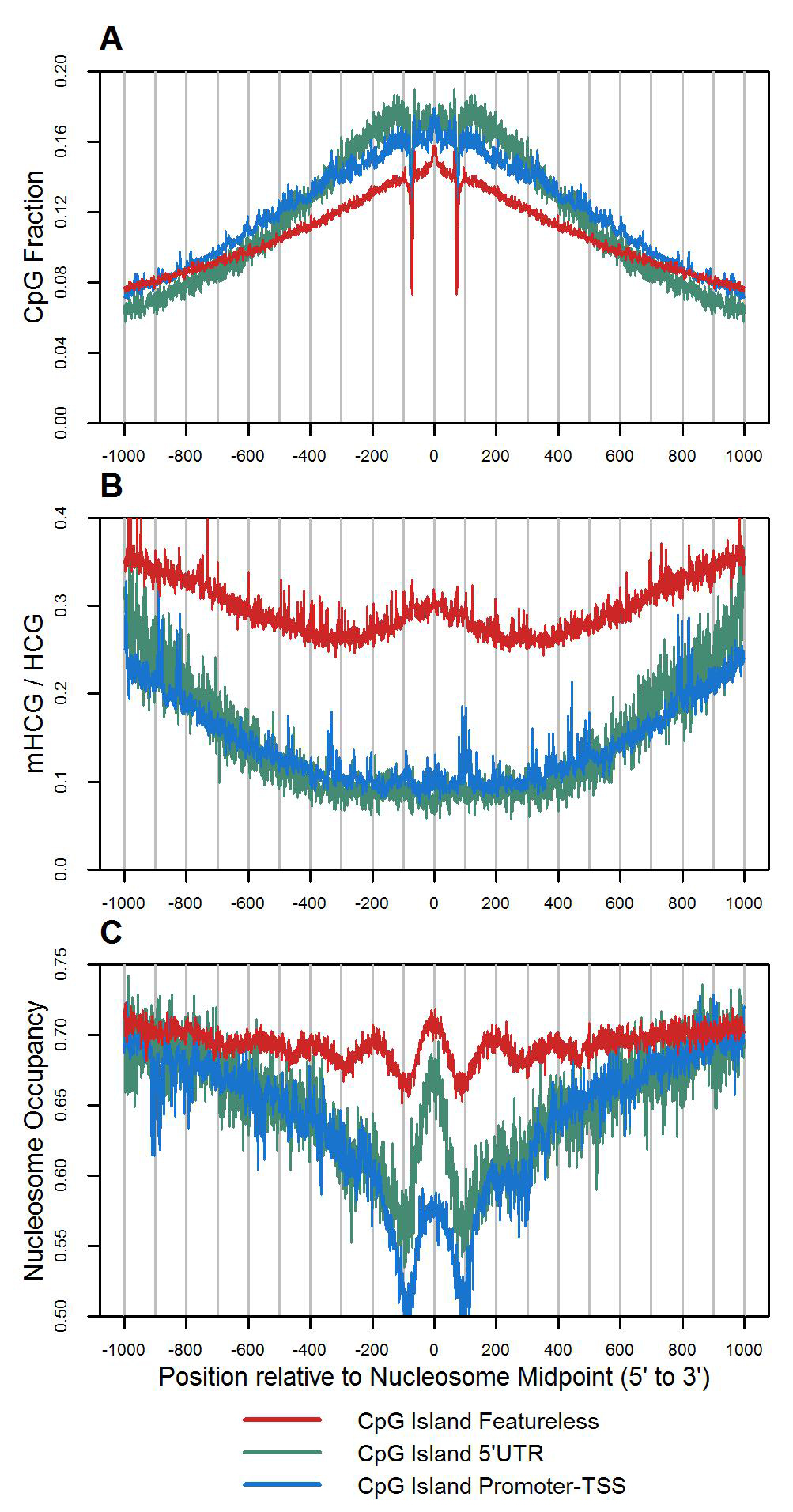


Figure S7. Frequency profiles of CpGs, DNA methylation levels, and nucleosome occupancy surrounding nucleosomes positioned within CpG Islands as annotated by the HOMER software. Nucleosomes in CpG islands were divided into three groups: promoter-TSS CpGIs, 5’UTR CpGIs, and ‘featureless’ CpGIs. Promoter-TSS regions are defined as -1000 to +100 relative to transcription start sites. Using MNase-seq and NOMe-seq data from IMR90 cells, average occurrences of CpGs (A), mHCG/HCG fractions (B) and uGCH/GCH fractions (C) were computed from forward and reverse complement sequences aligned to MNase-seq derived nucleosome midpoints within these features.


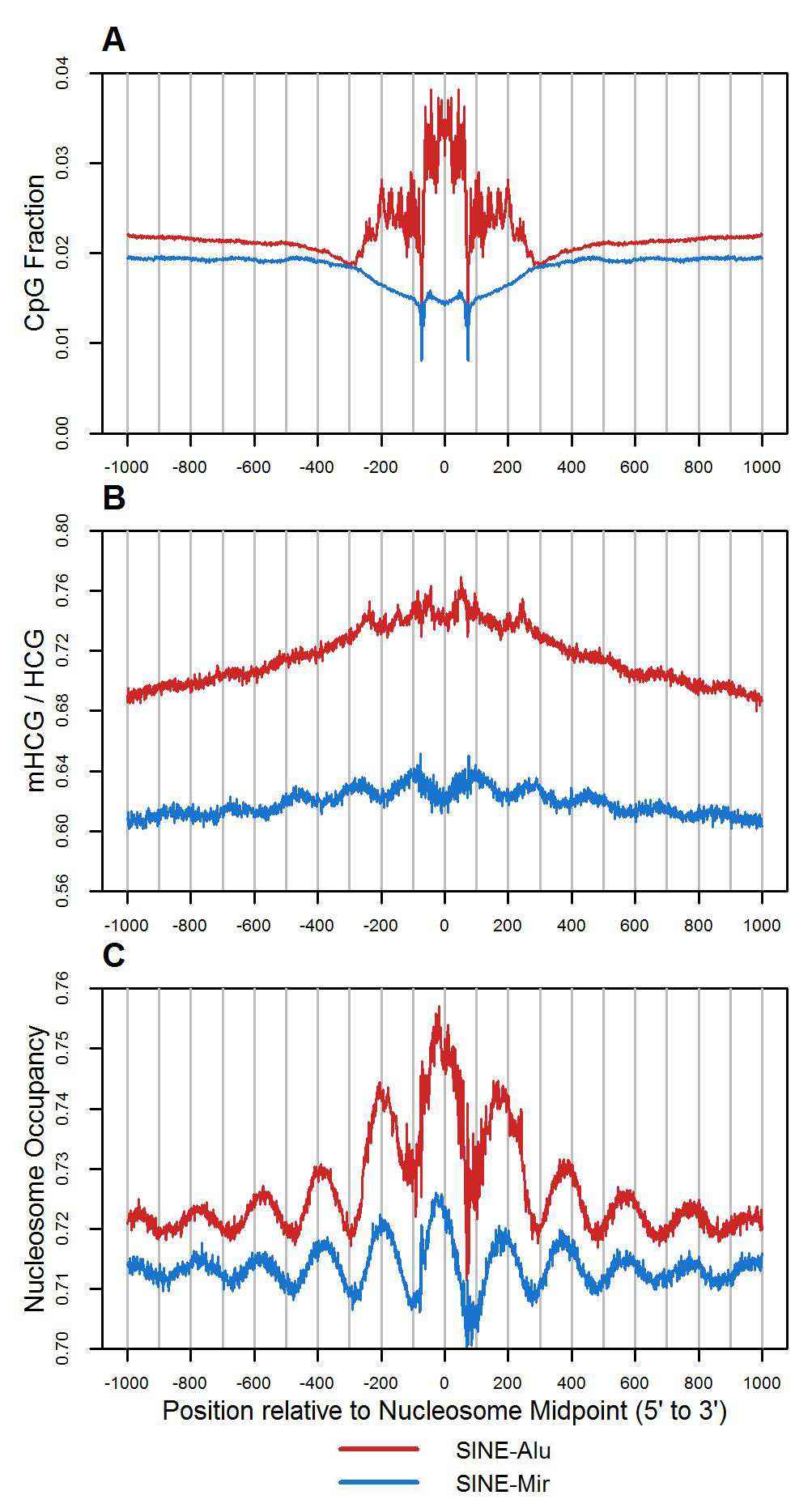


Figure S8. Frequency profiles of CpGs, DNA methylation levels, and nucleosome occupancy surrounding nucleosomes positioned within SINE-Alu and SINE-Mir elements as annotated by the HOMER software. Using MNase-seq and NOMe-seq data from IMR90 cells, average occurrences of CpGs (A), mHCG/HCG fractions (B) and uGCH/GCH fractions (C) were computed from forward and reverse complement sequences aligned to MNase-seq derived nucleosome midpoints within these features.


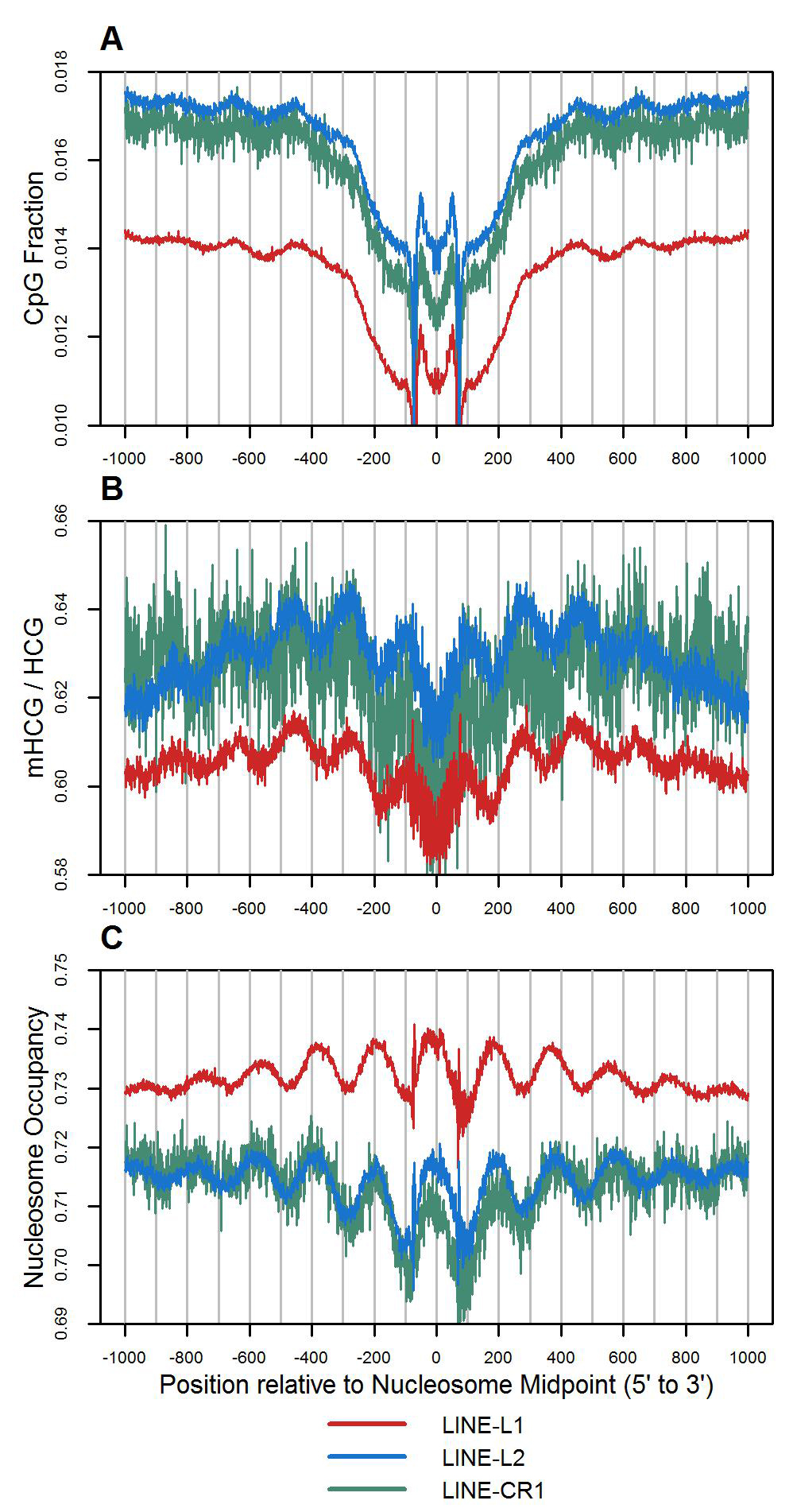


Figure S9. Frequency profiles of CpGs, DNA methylation levels, and nucleosome occupancy surrounding nucleosomes positioned within LINE-L1, LINE-L2, and LINE-CR1 elements as annotated by the HOMER software. Using MNase-seq and NOMe-seq data from IMR90 cells, average occurrences of CpGs (A), mHCG/HCG fractions (B) and uGCH/GCH fractions (C) were computed from forward and reverse complement sequences aligned to MNase-seq derived nucleosome midpoints within these features.


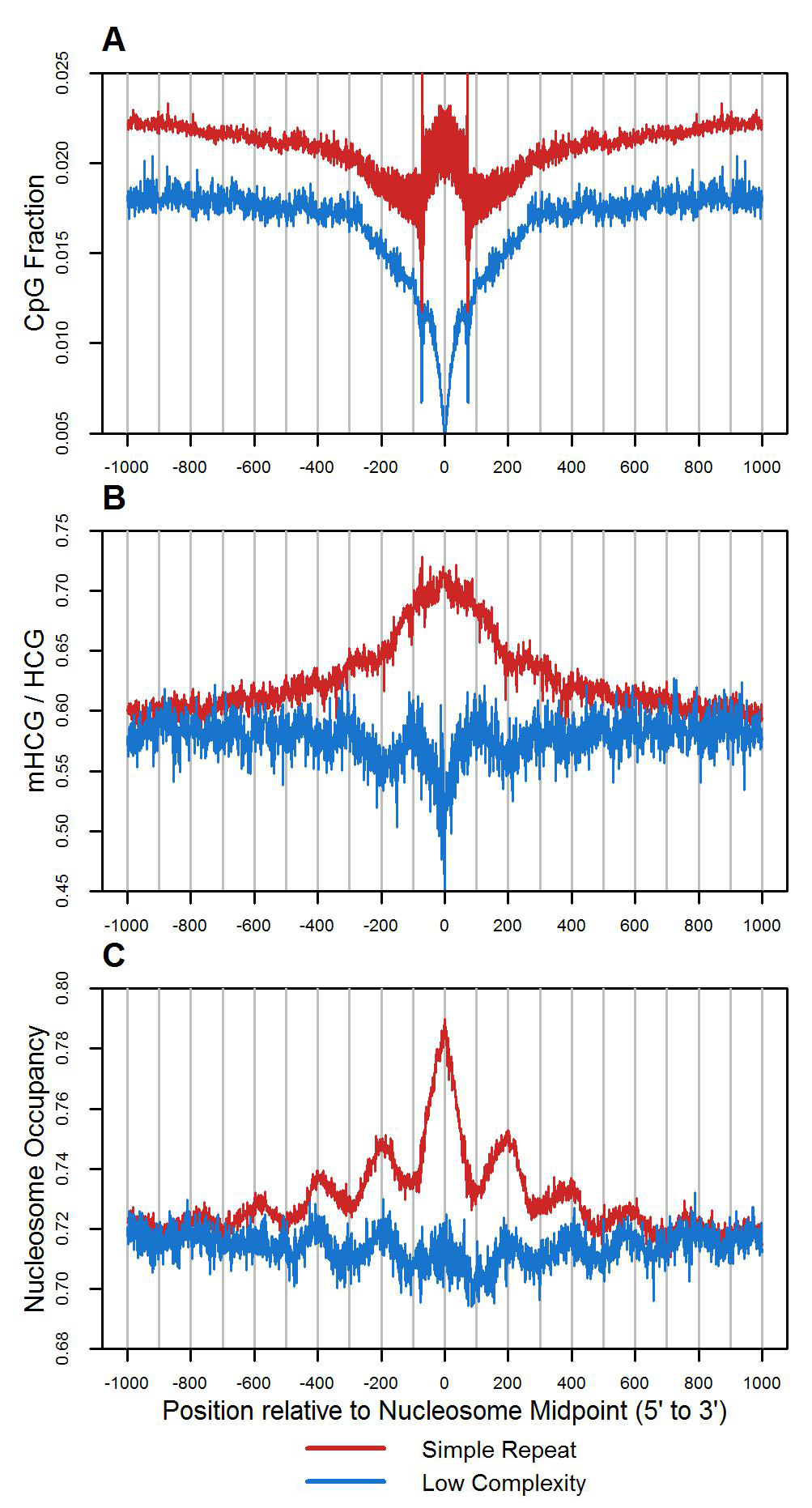


Figure S10. Frequency profiles of CpGs, DNA methylation levels, and nucleosome occupancy surrounding nucleosomes positioned within simple repeat and low complexity regions as annotated by the HOMER software. Using MNase-seq and NOMe-seq data from IMR90 cells, average occurrences of CpGs (A), mHCG/HCG fractions (B) and uGCH/GCH fractions (C) were computed from forward and reverse complement sequences aligned to MNase-seq derived nucleosome midpoints within these features.


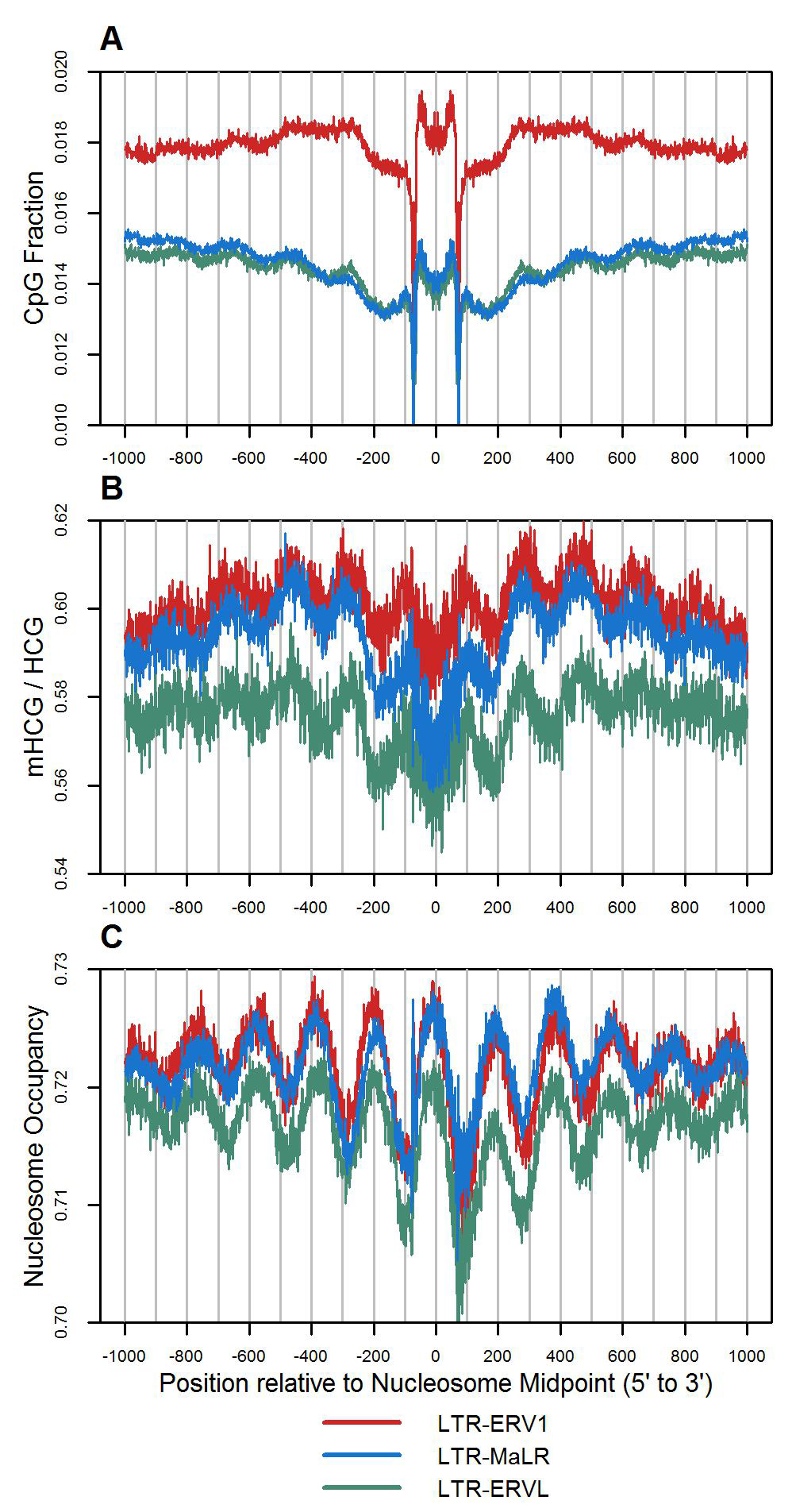


Figure S11. Frequency profiles of CpGs, DNA methylation levels, and nucleosome occupancy surrounding nucleosomes positioned within LTR-ERV1, LTR-MaLR, and LTR-ERVL elements as annotated by the HOMER software. Using MNase-seq and NOMe-seq data from IMR90 cells, average occurrences of CpGs (A), mHCG/HCG fractions (B) and uGCH/GCH fractions (C) were computed from forward and reverse complement sequences aligned to MNase-seq derived nucleosome midpoints within these features.


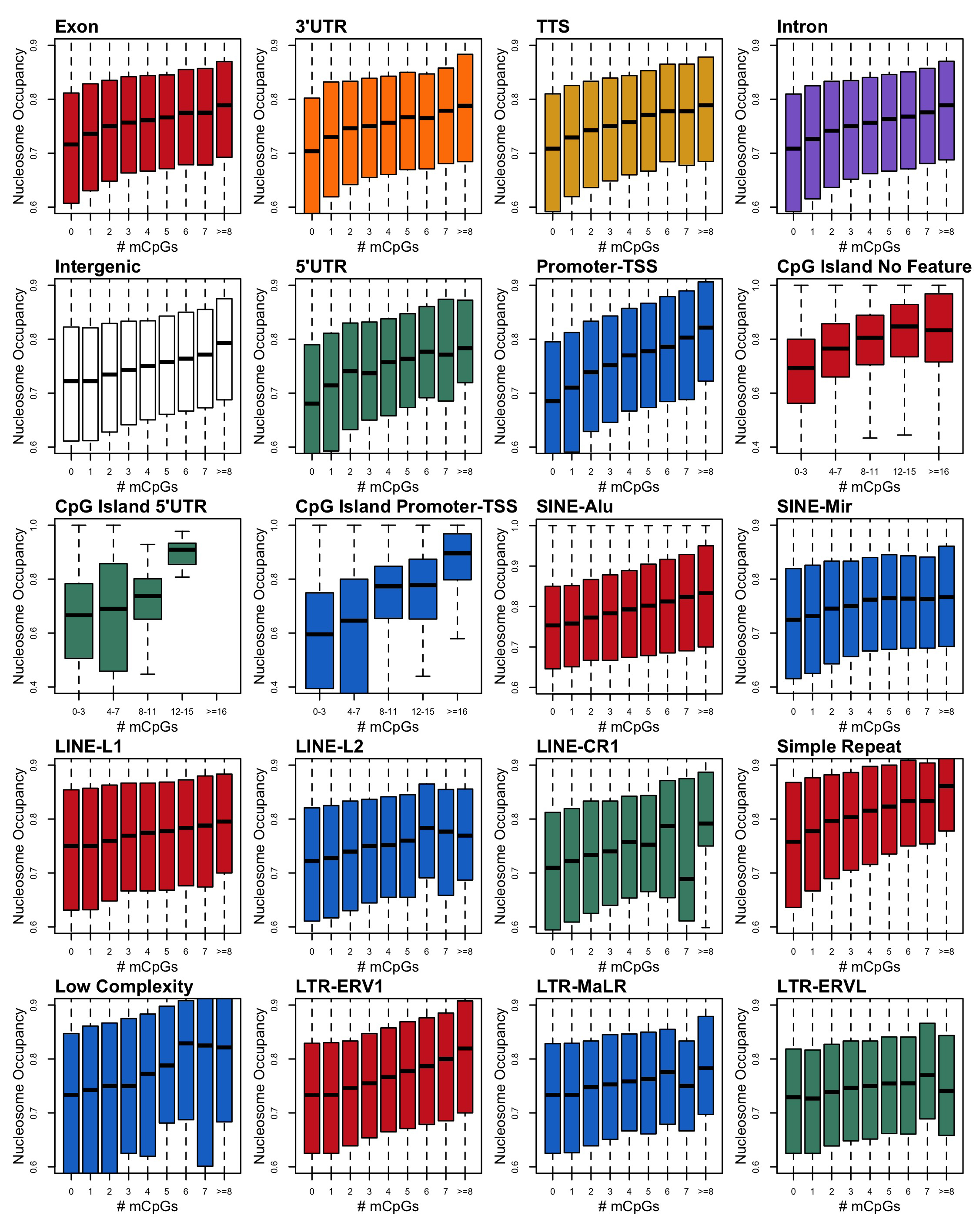


Figure S12. Effects of increasing methylated CpG density in the nucleosome core on nucleosome occupancy in individual features. The same procedure described for Figure 3 was carried out for nucleosomes positioned in the features indicated in the figure. With these data, boxplots were used to display the distribution of nucleosome occupancy as a function of the number of methylated CpGs. The colors of the boxplots for each feature are based on the colors used in Figures S6-S11. The nucleosome occupancy scales are the same for all panels except for the SINE-Alu one and CpG Island panels.


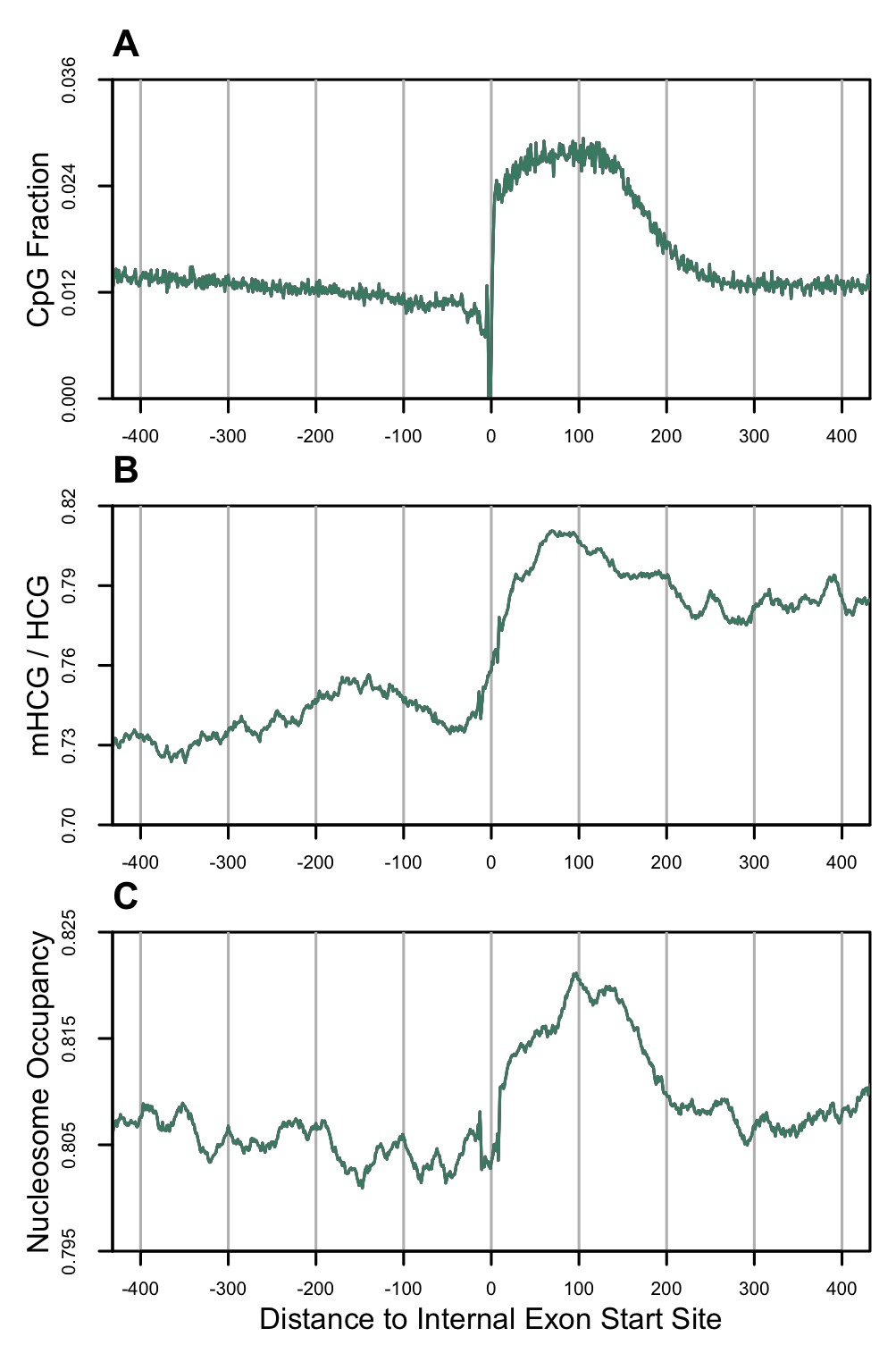

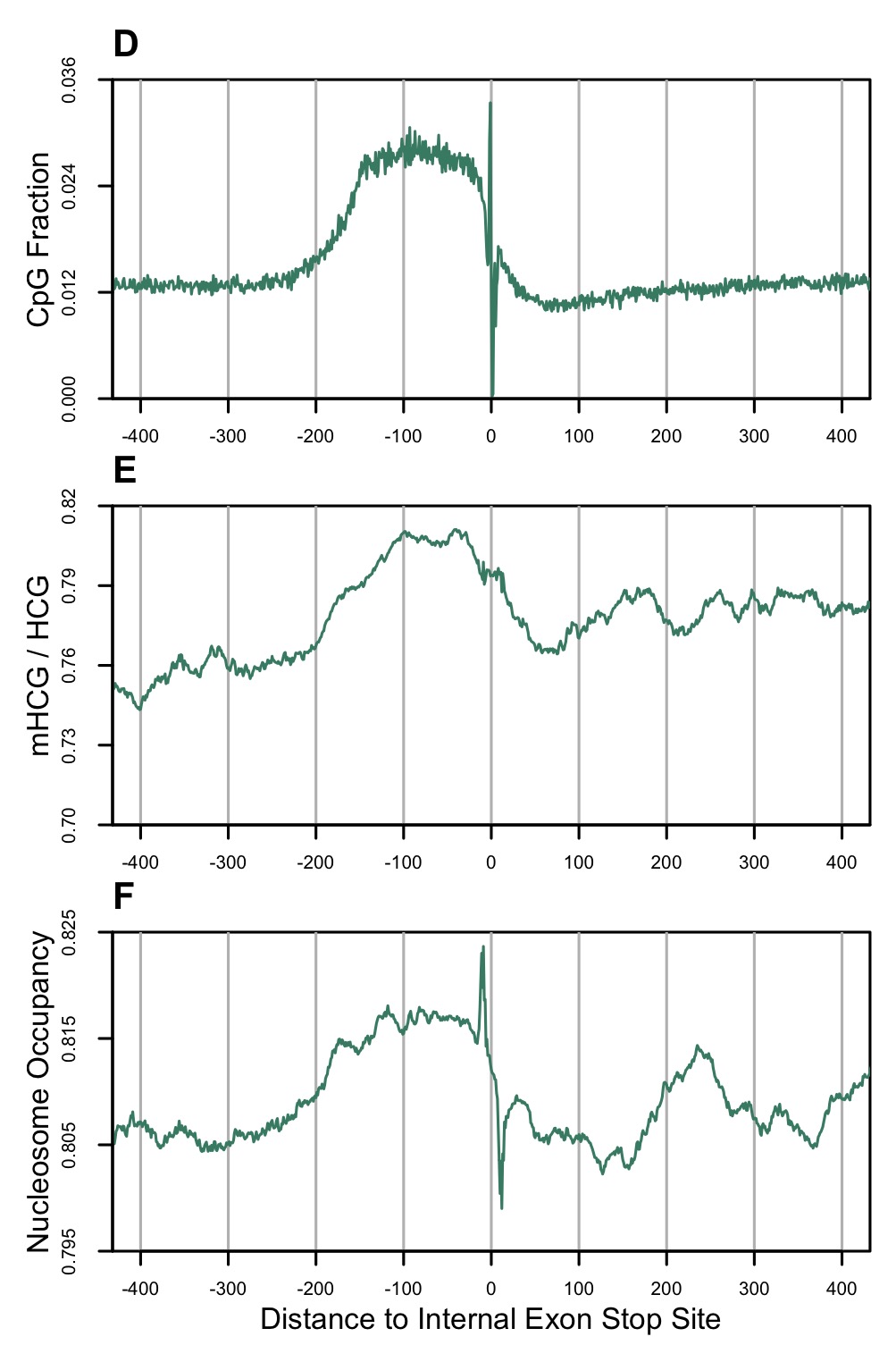


Figure S13. Frequency profiles of CpGs, DNA methylation levels, and nucleosome occupancy relative to internal exon start and stop sites. Using NOMe-seq data from IMR90 cells, average occurrences of CpGs (A,D), mHCG/HCG fractions (B,E) and uGCH/GCH fractions (C,F) were computed from forward and reverse complement sequences aligned to internal exon start and stop sites. Profiles of mHCG/HCG fractions and uGCH/GCH fractions were smoothed using 21 bp moving averages.


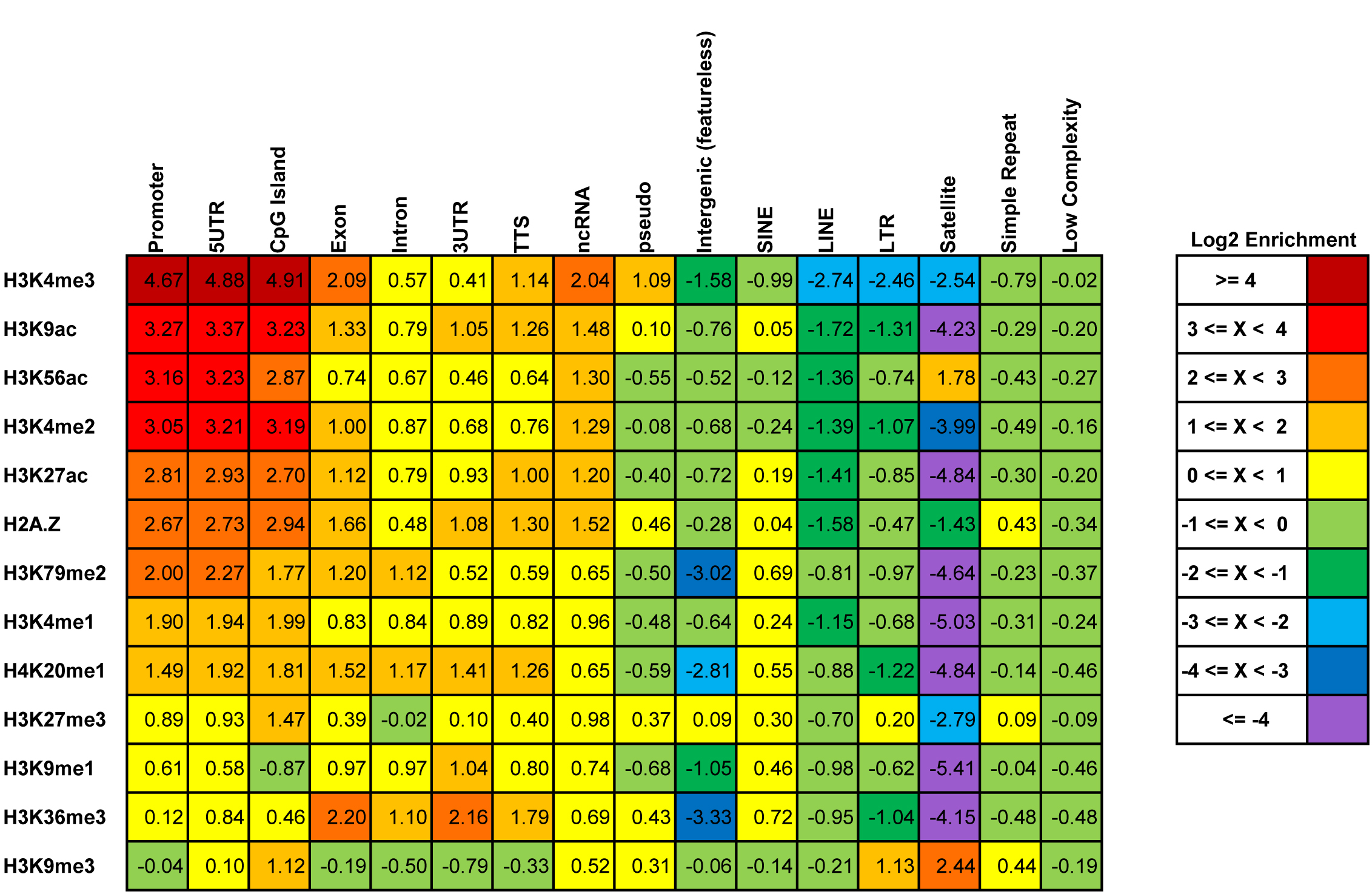


Figure S14. Annotation of histone modification domains in IMR90 cells. Each base pair in every peak called by SICER for the 12 histone modifications and the histone variant H2A.Z in the figure was annotated by the HOMER software. Log2 enrichments for each histone modification in the given features were determined and presented in the level plot.


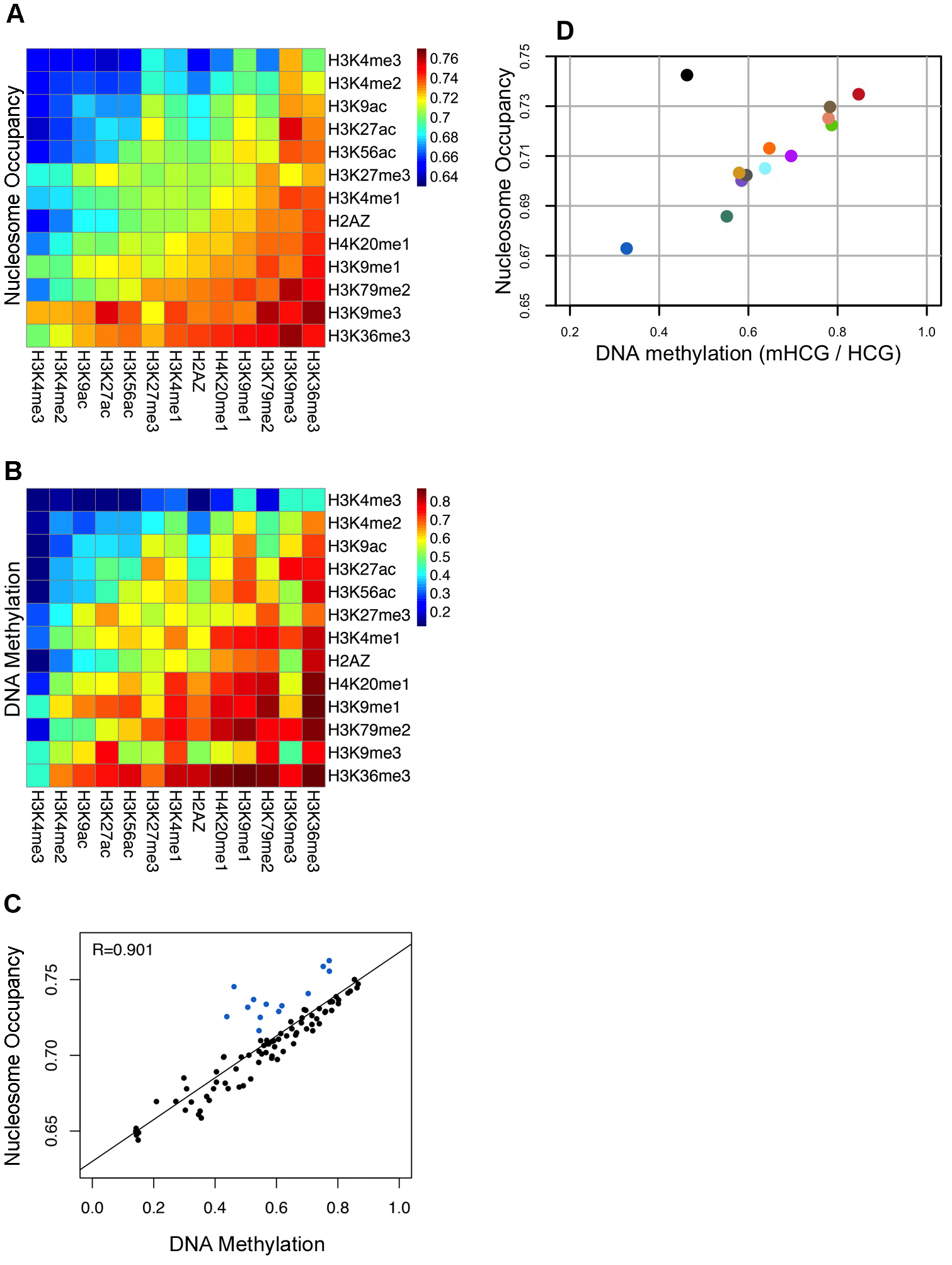


Figure S15. Comparison of nucleosome occupancy and DNA methylation in differentially marked chromatin across the genome. The same data for panels E and F from Figure 5 are shown except the data in panel F in this figure display average DNA methylation values instead of numbers of mCpGs per nucleosome. In panel C, the data from panels and A and B are plotted and the correlation coefficient is given in the figure. Blue dots represent histone modifications paired with H3K9me3. The same data for panel D from Figure 5D are shown except the values on the x axis represent average DNA methylation values instead of numbers of mCpGs per nucleosome. The same positive correlation in Figure 5D is observed for all histone modification except once again, H3K9me3 (black dot) is an outlier.


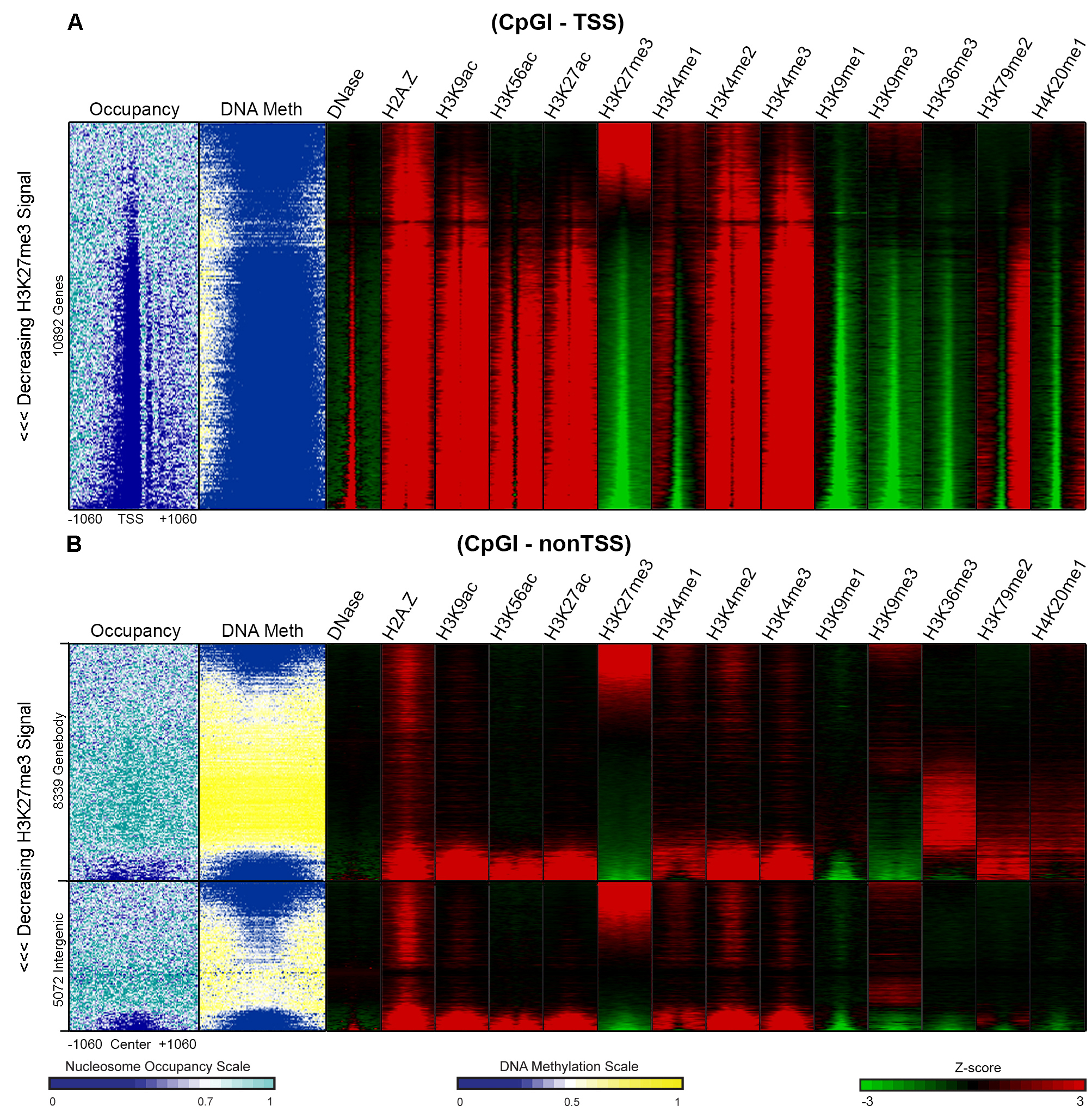


Figure S16. Characterization of chromatin at CpG islands sorted by decreasing H3K27me3. Using NOMe-seq and Roadmap ChIP-seq data from IMR90 cells, nucleosome occupancy, DNA methylation levels, and signals from 12 histone modifications and the histone variant H2A.Z were aligned to TSSs that overlapped CpG Islands (A) and to the centers of CpG islands located in genebodies and intergenic regions (B). In the heatmaps, average Z-scores were computed in 101 21bp bins surrounding the TSSs and nonTSS CpG island centers and were sorted by decreasing H3K27me3 occupancy.


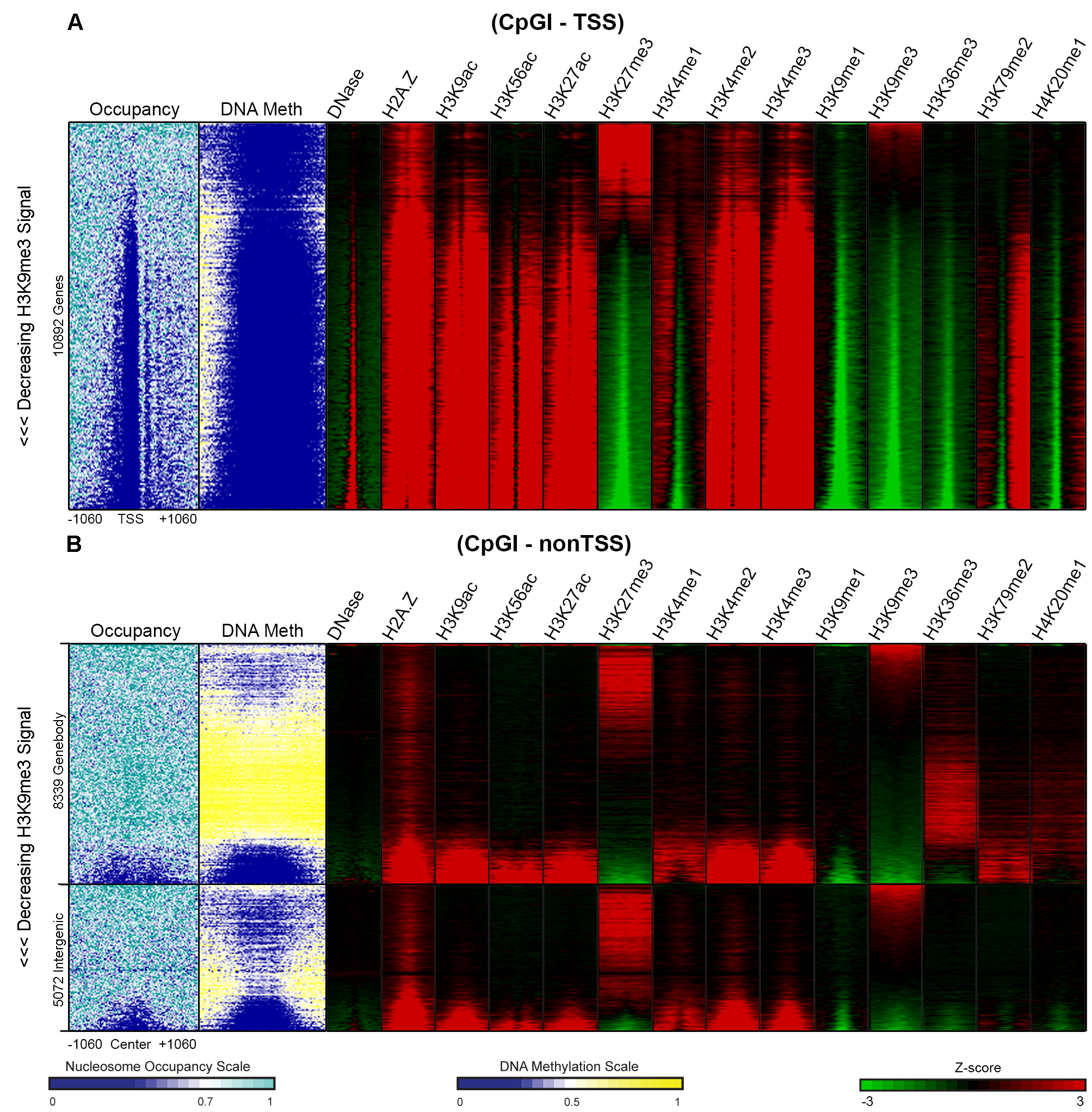


Figure S17. Characterization of chromatin at CpG islands sorted by decreasing H3K9me3. Using NOMe-seq and Roadmap ChIP-seq data from IMR90 cells, nucleosome occupancy, DNA methylation levels, and signals from 12 histone modifications and the histone variant H2A.Z were aligned to TSSs that overlapped CpG Islands (A) and to the centers of CpG islands located in genebodies and intergenic regions (B). In the heatmaps, average Z-scores were computed in 101 21bp bins surrounding the TSSs and nonTSS CpG island centers and were sorted by decreasing H3K9me3 occupancy.


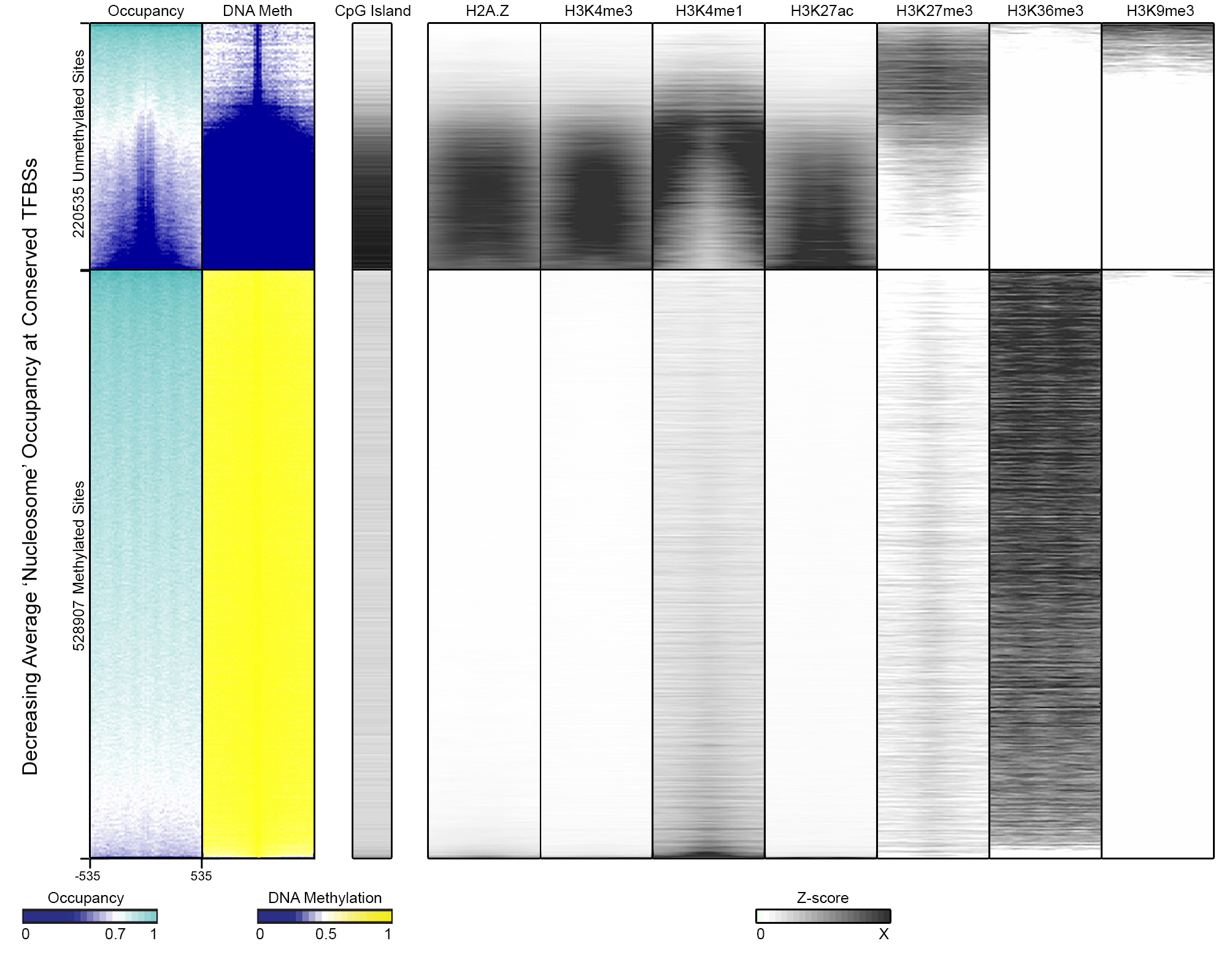


Figure S18. Extended characterization of chromatin at unmethylated and methylated conserved transcription factor binding sites. The same NOMe-seq and BS-seq data from HCT116 cells presented in Figure 7 are displayed in the figure along with ChIP-seq data from Lay et al. 2015 for 6 histone modifications and the histone variant H2A.Z. Additionally, a binary heatmap indicating whether or not a conserved TFBS overlapped a CpG island is shown. Signals for the histone modifications are represented by Z-scores. X = 4 for H2A.Z, 12 for H3K4me3, 2 for H3K4me1, 10 for H3K27ac, 0.8 for H3K27me3, 0.2 for H3K36me3, and 0.1 for H3K9me3.


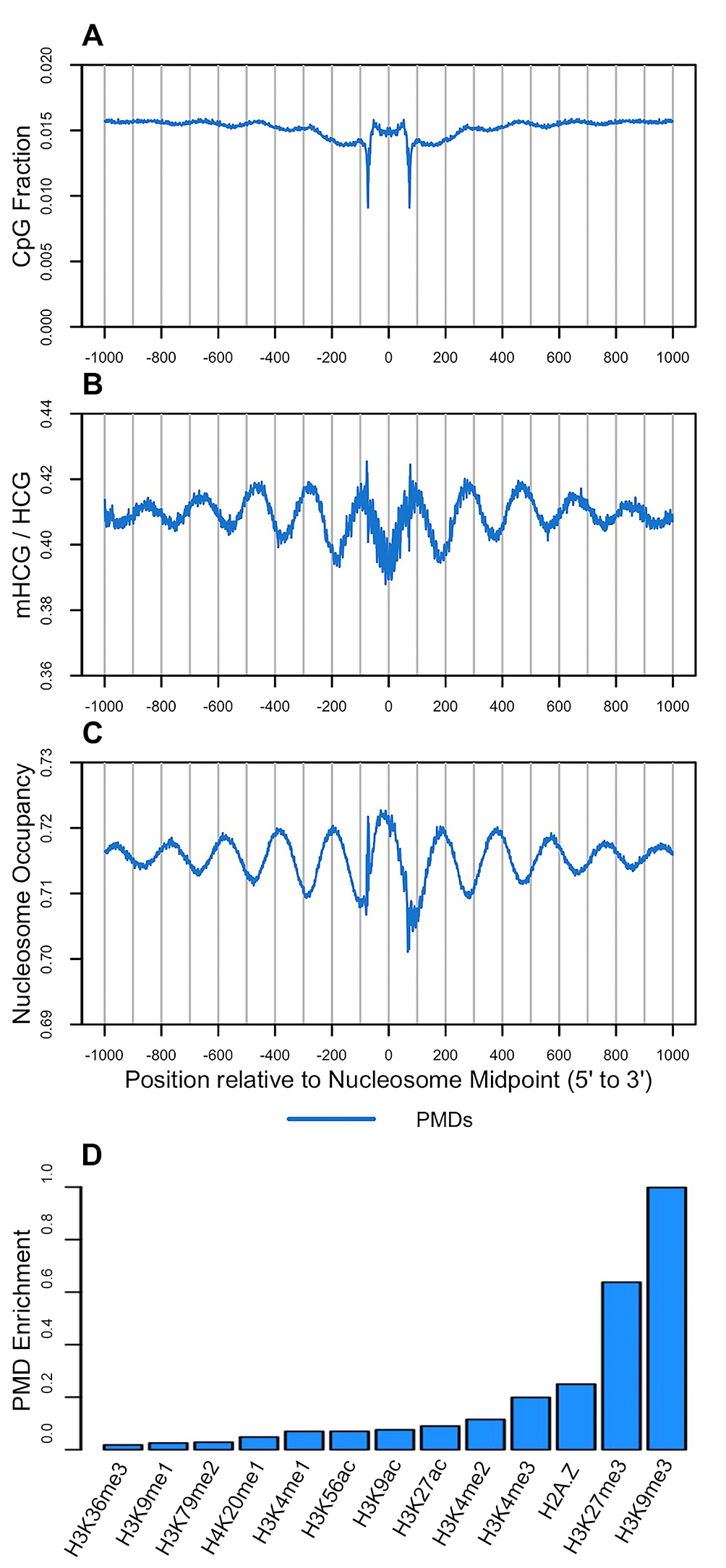


Figure S19. Frequency profiles of CpGs, DNA methylation levels, and nucleosome occupancy surrounding nucleosomes positioned within partially methylated domains. Using MNase-seq and NOMe-seq data from IMR90 cells, average occurrences of CpGs (A), mHCG/HCG fractions (B) and uGCH/GCH fractions (C) were computed from forward and reverse complement sequences aligned to MNase-seq derived nucleosome midpoints within partially methylated domains. D) Enrichment of histone modifications in PMDs were determined by calculating the following ratio: the base pair overlap of peaks called by SICER with PMD coordinates divided by the total SICER peak length over the total PMD length divided by the length of the human genome.
