# Supplementary figures and images for: Links between DNA methylation and nucleosome occupancy in the human genome
Source: Epigenetics Chromatin. 2017 Apr 11;10:18. doi: 10.1186/s13072-017-0125-5 (PMC5387343; doi:10.1186/s13072-017-0125-5)

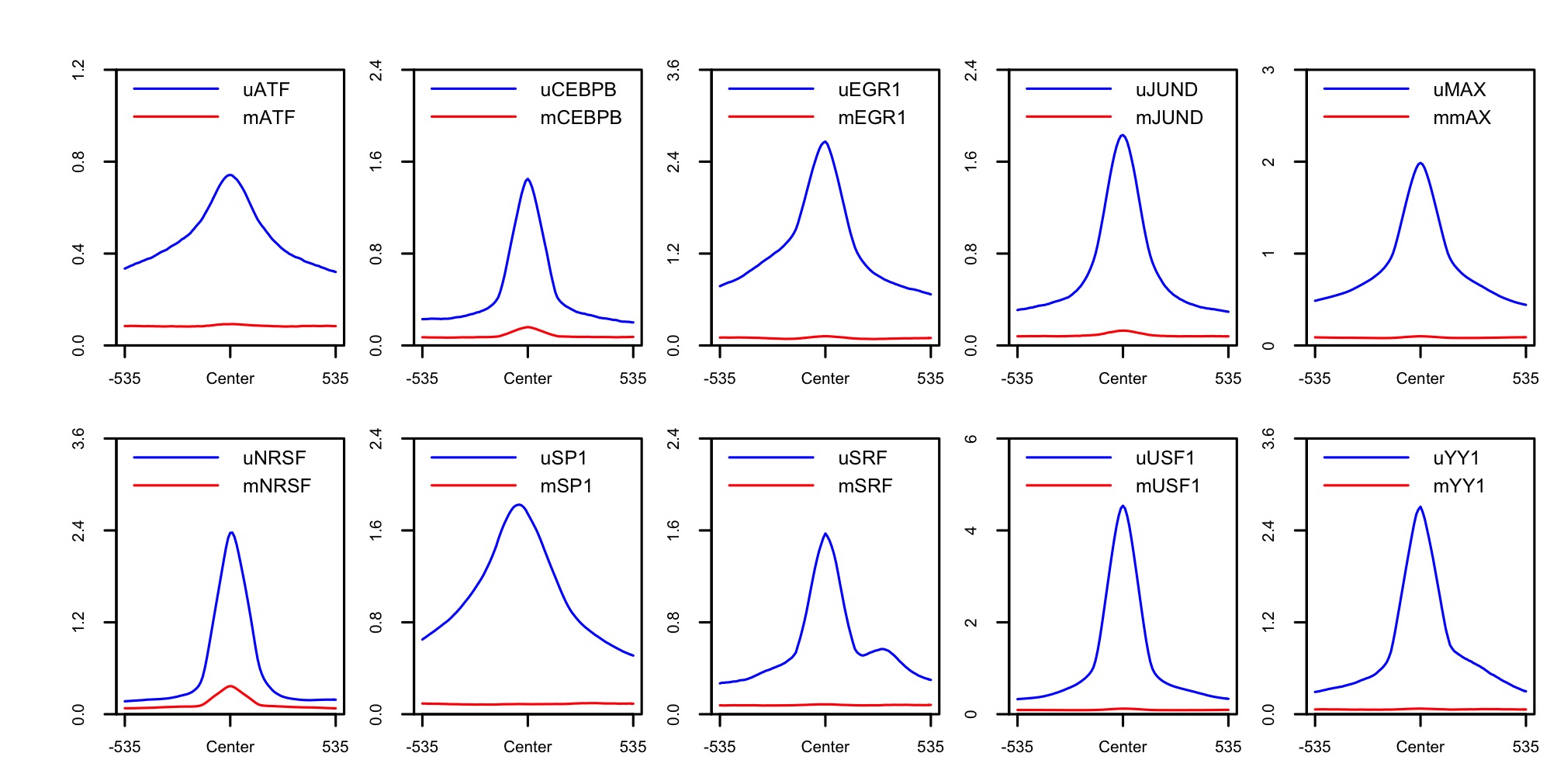

Supplement: Supplementary file 2 — Additional file 2. Detailed bioinformatics procedures and in-house scripts used in this study are enclosed in the zip file. [file 13072_2017_125_MOESM2_ESM.zip › Supplmentary.Methods.2017.0320/Figure7.FigureS18/figure7D.WGBS.jpeg]
