# Supplementary material for: Links between DNA methylation and nucleosome occupancy in the human genome
Source: Epigenetics Chromatin. 2017 Apr 11;10:18. doi: 10.1186/s13072-017-0125-5 (PMC5387343; doi:10.1186/s13072-017-0125-5)
Supplement: Supplementary file 2 — Additional file 2. Detailed bioinformatics procedures and in-house scripts used in this study are enclosed in the zip file. [file 13072_2017_125_MOESM2_ESM.zip › Supplmentary.Methods.2017.0320/Supplementary.Methods.docx]

**Supplementary Methods**

Note: Several of the provided perl, R, and shell scripts described in this document are uncommented and may require additional steps or modifications to be utilized on different computational platforms. For example, some scripts use large amounts of RAM or require supplemental software packages. Feel free to contact us if you have questions or need assistance.

1) **Figures 1 and 2 – DNA methylation and Nucleosome Occupancy at the Genome Level**

**(includes some data acquisition, processing, and annotation steps)**

A. **Leukocyte Data**
1. downloaded processed MNase-seq data (hg18)

sh getFireProcessed.sh

2. converted *in vivo*, *in vitro, and* MNase control processed files to bed format and divided the data by chromosome

perl makeChrBedFire.pl InVivo/

perl makeChrBedFire.pl InVitro/

perl makeChrBedFire.pl MNase_Control/

3. used HOMER’s ‘annotatePeaks.pl’ script to annotate each nucleosome at its midpoint

perl annotatePeaks.pl Leukocyte.reads/InVitro_New_chr1.bed hg18 -annStats Leukocyte.annotation_stats/annotation_stats_InVitro_New_chr1.txt > Leukocyte.annotated_reads/annotated_InVitro_New_chr1.txt

replicated for all chromosomes for all three datasets

4. normalized *in vivo* and *in vitro data* and subtracted normalized MNase-seq control data as background from the *in vivo* and *in vitro* data on the annotated text files separated by chromosome

perl subtractControlInVitro.pl Leukocyte.annotation_reads/annotated_InVitro_New_chr1.txt Leukocyte.annotation_reads/annotated_MNase_Control_New_chr1.txt chr1

perl subtractControlInVivo.pl Leukocyte.annotation_reads/annotated_InVivo_New_chr1.txt annotation_reads/annotated_MNase_Control_New_chr1.txt chr1

replicated for all chromosomes for *in vivo* and *in vitro* datasets

5. downloaded BS-seq data (hg19), used UCSC ‘bigBedToBed’ tool, converted coordinates from hg19 to hg18 with UCSC liftover tool, and divided the data into files by chromsome

wget http://www.ebi.ac.uk/arrayexpress/files/E-GEOD-40832/E-GEOD-40832.processed.2.zip/GSM1002650_hg19_wgEncodeHaibMethylWgbsGm12878CpGSites.bigBed

bigBedToBed GSM1002650_hg19_wgEncodeHaibMethylWgbsGm12878CpGSites.bigBed hg19_gm12878_wgbs.bed

liftOver -bedPlus=10 hg19_gm12878_wgbs.bed hg19ToHg18.over.chain hg18_gm12878_wgbs.bed hg19_gm12878_wgbs.unmapped.bed

awk '{print $0 >> "hg18_gm12878_wgbs_"$1".bed"}' hg18_gm12878_wgbs.bed

6. quantified occurrences of dinucleotides surrounding all nucleosomes in the genome by chromosome for the *in vivo* and *in vitro* datasets outside of CpG islands

perl makeInVitroNucMethProfilesForMonoDiMethByChrWithoutCpGIs.pl hg18_gm12878_wgbs_chr1.bed hg18/chr1.fa InVitro.norm.anno.reads/InVitro_normalized_subtract_control_chr1.txt hg18_CpG_Islands.txt chr1

perl makeInVivoNucMethProfilesForMonoDiMethByChrWithoutCpGIs.pl hg18_gm12878_wgbs_chr1.bed hg18/chr1.fa InVivo.norm.anno.reads/InVivo_normalized_subtract_control_chr1.txt hg18_CpG_Islands.txt chr1

replicated for all chromosomes for *in vivo* and *in vitro* datasets

7. combined chromosomal *in vitro* data for Figures 1A, 1B, 1E, and 1F.

perl getInVitroDiProfiles.pl InVitroDi/

perl getInVitroMethProfiles.pl InVitroMeth/

8. combined chromosomal *in vivo* data for Figure 1B, 1E, and 1F

perl getInVivoDiProfiles.pl InVivoDi/

perl getInVivoMethProfiles.pl InVivoMeth/

9. used mCpG/CpG fractions and dinucleotide occurances to determine values for Figures 1C & 1D
10. quantified *in vitro* data by chromosome for Figure 2A

perl makeInVitroNucMethProfilesCpGcounts.pl hg18_gm12878_wgbs_chr1.bed hg18/chr1.fa InVitro_normalized_subtract_control_chr1.txt hg18_CpG_Islands.txt chr1

replicated for remaining chromosomes

11. combined chromosomal *in vitro* data for Figure 2A

getInVitroCpGCountMethProfiles.pl InVitro_CpG_Counts/

12. quantified *in vivo* data by chromosome for Figure 2B

perl makeInVivoNucMethProfilesCpGcounts.pl hg18_gm12878_wgbs_chr1.bed hg18/chr1.fa InVivo_normalized_subtract_control_chr1.txt hg18_CpG_Islands.txt chr1

replicated for remaining chromosomes

13. combined chromosomal *in vivo* data for Figure 2B

getInVivoCpGCountMethProfiles.pl InVivo_CpG_Counts/

Primary Output Files: invivo_di.txt, invitro_di.txt, invivo_meth.txt, invitro_meth.txt

Primary Output Folders: InVivo_CpG_Counts/, InVitro_CpG_Counts/

B. **IMR90 data**
14. downloaded processed MNase-seq data (hg18), merged bam files, sorted merged bam file, and converted bam file to bed format

wget ftp://ftp.ncbi.nlm.nih.gov/geo/samples/GSM543nnn/GSM543311/suppl/ GSM543311_ResultCount_*.bam

sh mergeIMR90bam2bed.sh

15. converted *in vivo* MNase-seq reads to same bed format used in step 2 and separated the data by chromosome

perl makeChrBedIMR90.pl IMR90_MNase-seq.sorted.bed

16. used HOMER’s ‘annotatePeaks.pl’ script to annotate each nucleosome at its midpoint

perl annotatePeaks.pl IMR90.reads/IMR90_MNase-seq_chr1.bed hg18 -annStats IMR.90.annotation_stats/annotation_stats_IMR90_MNase-seq_chr1.txt > IMR90.annotated_reads/annotated_ IMR90_MNase-seq_chr1.txt

replicated for all chromosomes

17. downloaded NOMe-seq data and divided NOMe-seq data by chromosome

wget ftp://ftp.ncbi.nlm.nih.gov/geo/samples/GSM1001nnn/GSM1001125/suppl/ GSM1001125_IMR90.hg18.gch.wig.gz

wget ftp://ftp.ncbi.nlm.nih.gov/geo/samples/GSM1001nnn/GSM1001125/suppl/ GSM1001125_IMR90.hg18.hcg.wig.gz

gunzip *.gz

mv GSM1001125_IMR90.hg18.gch.wig nomeseq_imr90_gch.wig

mv GSM1001125_IMR90.hg18.hcg.wig nomeseq_imr90_hcg.wig

perl divide_nomeseq_wig.pl nomeseq_imr90_gch.wig nomeseq_imr90_hcg.wig

18. quantified DNA methylation and nucleosome occupancy levels surrounding all nucleosomes in the genome for each chromosome for several annotated features

perl makeNOMeSeqMethProfilesFeaturesAll.pl nomeseq_imr90_hcg_chr1.txt nomeseq_imr90_gch_chr1.txt hg18/chr1.fa IMR90.annotated_reads/annotated_IMR90_MNase-seq_chr1.txt hg18_CpG_Islands.txt chr1

replicated for all chromosomes

19. combined chromosomal NOMe-seq DNA methylation data for Figures 1G and 1I

perl getHCG.AllProfiles.pl NOMe-seq_Features/

20. combined chromosomal NOMe-seq nucleosome occupancy data for Figures 1H and 1J

perl getGCH.AllProfiles.pl NOMe-seq_Features/

21. quantified NOMe-seq data by chromosome for Figures 2C and 2D

perl makeNOMe-seqNucMethProfilesCpGcounts.pl nomeseq_imr90_hcg_chr1.txt nomeseq_imr90_gch_chr1.txt hg18/chr1.fa IMR.90.annotated_reads/annotated_IMR90_MNase-seq_chr1.txt hg18_CpG_Islands.txt chr1

replicated for all chromosomes

22. combined chromosomal NOMe-seq DNA methylation data for Figure 2C

getNOMeSeqCpGCountHCGProfiles.pl NOMe-seq_CpG_Counts/

23. combined chromosomal NOMe-seq nucleosome occupancy data for Figure 2D

getNOMeSeqCpGCountGCHProfiles.pl NOMe-seq_CpG_Counts/

Primary Output Files: nomeseq_all_GCH.txt, nomeseq_all_HCG.txt

Primary Output Folders: NOMe-seq_CpG_Counts/, NOMe-seq_Features/

C. **Figure Generation**
24. used output files above to assemble figure1.txt and figure2.txt
25. used R codes and photoshop to generate figures 1 and 2

Rscript figure1.code.R

Rscript figure2.code.R

Primary Output Files: figure1.jpg, figure2.jpg

2) **Figure S2 and S3 – Effects of Increasing Linker CpG Density**
26. quantified quantified *in vitro* data by chromosome for Figure S2A

perl makeInVitroNucMethProfilesCpGcounts.Linker.pl hg18_gm12878_wgbs_chr1.bed hg18/chr1.fa InVitro_normalized_subtract_control_chr1.txt hg18_CpG_Islands.txt chr1

replicated for remaining chromosomes

27. combined chromosomal *in vitro* data for Figure S2A

getInVitroCpGCountMethProfiles.Linker.pl InVitro_CpG_Counts.Linker/

28. quantified *in vivo* data by chromosome for Figure 2B

perl makeInVivoNucMethProfilesCpGcounts.Linker.pl hg18_gm12878_wgbs_chr1.bed hg18/chr1.fa InVivo_normalized_subtract_control_chr1.txt hg18_CpG_Islands.txt chr1

replicated for remaining chromosomes

29. combined chromosomal *in vivo* data for Figure S2B

getInVivoCpGCountMethProfiles.Linker.pl InVivo_CpG_Counts.Linker/

30. quantified NOMe-seq data by chromosome for Figures S2C, S2D, and S3

perl makeNOMe-seqNucMethProfilesCpGcountsLinker.pl nomeseq_imr90_hcg_chr1.txt nomeseq_imr90_gch_chr1.txt hg18/chr1.fa IMR.90.annotated_reads/annotated_IMR90_MNase-seq_chr1.txt hg18_CpG_Islands.txt chr1

replicated for all chromosomes

31. combined chromosomal NOMe-seq DNA methylation data for Figure S2C

getNOMeSeqCpGCountHCGProfiles.Linker.pl NOMe-seq_CpG_Counts.Linker/

32. combined chromosomal NOMe-seq nucleosome occupancy data for Figure S2D

getNOMeSeqCpGCountGCHProfiles.Linker.pl NOMe-seq_CpG_Counts.Linker/

33. combined chromosomal NOMe-seq nucleosome occupancy data for Figure S3A

getNOMeSeqCpGCountCGProfiles.Linker.pl NOMe-seq_CpG_Counts.Linker/

34. combined chromosomal NOMe-seq nucleosome occupancy data for Figure S3C

getNOMeSeqCpGCountCGProfiles.Nucleosome.pl NOMe-seq_CpG_Counts /

35. generated boxplot data for genome, exons, and intergenic nucleosomes

Note: did this after I completing Figure 3 below.

perl makeBoxPlotData.genome2.pl NOMeSeq.individual.nucleosome.and.linker.info/

Primary Output Files: nomeseq_cpg_count_gch_linker.txt, nomeseq_cpg_count_hcg_linker.txt, nomeseq.CpGcounts.mCpGprofiles.figure2.linker.txt, invitro.invivo.cpg.count.meth.5.linker.txt,

N_CG.genome.linker.txt, R_CG.genome.linker.txt

Primary Output Folders: NOMe-seq_CpG_Counts/Linker/, InVivo_CpG_Counts.Linker/, InVitro_CpG_Counts.Linker/

36. used output files to assemble figureS2S3.txt
37. used R codes and photoshop to generate figures S2 and S3

Rscript figureS2EF.code.R

Rscript figureS2S3.code.R

Primary Output Files: figureS2.jpg, figureS2.jpg

3) **Figure S1 - DNA methylation and Nucleosome Occupancy surrounding CTCF Sites**
A. **Leukocyte Data**
38. quantified DNA methylation levels surrounding CTCF sites

perl makeInVivoMethProfilesForCTCF.pl hg18/chr1.fa hg18_gm12878_wgbs_chr1.bed hg18_ctcf_comp_predicted_sites_13401.txt chr1

replicated for all chromosomes

39. combined chromosomal *in vivo* data for Figure S1B

perl getInVivoMethCTCFProfile.pl InVivo_CTCF/

Primary Output Files:	invivo_CTCF_meth.txt

B. **IMR90 data**
40. quantified DNA methylation and nucleosome occupancy levels surrounding CTCF sites

mkdir nomeseq_imr90_hcg

cp nomeseq_imr90_hcg* nomeseq_imr90_hcg/

mkdir nomeseq_imr90_gch

cp nomeseq_imr90_gch* nomeseq_imr90_gch/

perl makeNOMeSeqProfileForCTCF.pl nomeseq_imr90_hcg/ nomeseq_imr90_gch/ hg18/ hg18_ctcf_comp_predicted_sites_13401.txt

Primary Output Files:	CTCF_comp_predicted_sites_hg18_NOMeSeq_profiles.txt

C. **Sequence data**
41. quantified DNA methylation and nucleosome occupancy levels surrounding CTCF sites

perl makeCpGProfileForCTCF.pl hg18/ hg18_ctcf_comp_predicted_sites_13401.txt

Primary Output Files:	hg18_CTCF_comp_predicted_CpG_profiles.txt

D. **Figure Generation**
42. used output files above to assemble figureS1.txt
43. used R codes and photoshop to generate figures S1

Rscript figureS1.code.R

Primary Output Files: figureS1.jpg

4) **Figures 3, S4, and S5 - DNA methylation and Nucleosome Occupancy in Various Sequence Contexts (genome-wide and in exonic / intergenic chromatin)**
A. **Figure 3 (Genome)**
44. determined the number of CpGs, G+C content, average mHCG/HCG fraction, and average uGCH/GCH fraction were computed for each nucleosome outside of CpG islands between positions -61 and +61 relative to the MNase-seq derived nucleosome midpoint

perl nucleosome_definer_genome.pl nomeseq_imr90_hcg_chr1.txt nomeseq_imr90_gch_chr1.txt hg18/chr1.fa IMR.90.reads/IMR90_MNase-seq_chr1.txt hg18_CpG_Islands.txt chr1

replicated for all chromosomes

45. determined core:linker mCpG density ratios

perl linker_definer_genome.pl nomeseq_imr90_hcg_chr1.txt nomeseq_imr90_gch_chr1.txt hg18/chr1.fa NOMeSeq.individual.nucleosome.no.CpGI.info /chr1.NOMeSeq.individual.nucleosome.no.CpGI.info.genome.txt chr1

replicated for all chromosomes

B. **Figure S4 (Intergenic)**
46. obtained intergenic subset of data from step 32

perl getIntergenic.pl NOMeSeq.individual.nucleosome.no.CpGI.info /chr1_NOMeSeq_individual.nucleosome.no.CpGI.info.genome.txt Leukocyte.annotated_reads/annotated_IMR90_MNase-seq_chr1.txt

replicated for all chromosomes

47. determined core:linker mCpG density ratios for intergenic nucleosomes

perl linker_definer_intergenic.pl nomeseq_imr90_hcg_chr1.txt nomeseq_imr90_gch_chr1.txt hg18/chr1.fa NOMeSeq.individual.nucleosome.no.CpGI.info /chr1.NOMeSeq.individual.nucleosome.no.CpGI.info.intergenic.txt chr1

replicated for all chromosomes

C. **Figure S5 (Exon)**
48. obtained exonic subset of data from step 32

perl getExon.pl NOMeSeq.individual.nucleosome.no.CpGI.info /chr1_NOMeSeq_individual.nucleosome.no.CpGI.info.genome.txt Leukocyte.annotated_reads/annotated_IMR90_MNase-seq_chr1.txt

replicated for all chromosomes

49. determined core:linker mCpG density ratios for exonic nucleosomes

perl linker_definer_exon.pl nomeseq_imr90_hcg_chr1.txt nomeseq_imr90_gch_chr1.txt hg18/chr1.fa NOMeSeq.individual.nucleosome.no.CpGI.info /chr1.NOMeSeq.individual.nucleosome.no.CpGI.info.exon.txt chr1

replicated for all chromosomes

D. **Figure Generation**
50. generated boxplot data for genome, exons, and intergenic nucleosomes

perl makeBoxPlotData.genome.pl NOMeSeq.individual.nucleosome.no.CpGI.info/

NOMeSeq.individual.nucleosome.and.linker.info/

perl makeBoxPlotData.intergenic.pl NOMeSeq.individual.nucleosome.no.CpGI.info/

NOMeSeq.individual.nucleosome.and.linker.info/

perl makeBoxPlotData.exon.pl NOMeSeq.individual.nucleosome.no.CpGI.info/

NOMeSeq.individual.nucleosome.and.linker.info/

51. used R codes and a little photoshop to produce figures 3, S4, and S5

Rscript figure3.code.R

Rscript figureS4.code.R

Rscript figureS5.code.R

Primary Output Folders: NOMeSeq.individual.nucleosome.no.CpGI.info/ NOMeSeq.individual.nucleosome.and.linker.info/

Primary Output Files: figure3.jpg, figureS4.jpg, figureS5.jpg

5) **Figures 4, and S6-S13 - DNA methylation and Nucleosome Occupancy in a Variety of Annotated Features**
A. **Figure 4 and Figures S6-S11**
52. determined the average CpG, mHCG/HCG, and uGCH/GCH fractions relative to the MNase-seq derived nucleosome midpoint in a variety of annotated features

perl getGCH.FeatureProfiles.pl NOMe-seq_Features/

perl getHCG.FeatureProfiles.pl NOMe-seq_Features/

perl getCG.FeatureProfiles.pl NOMe-seq_Features/

Primary Output Files: nomeseq_features_CG.txt, nomeseq_features_GCH.txt, nomeseq_features_HCG.txt

53. renamed columns in output files from Step 40 according to instructions in R code below and the R code and a little photoshop to produce figures 4 and S6-S11

Rscript figure4.S6-S11.code.R

Primary Output Files: figures 4 and S6-S11 and their legends in jpg format

B. **Figure S12**
54. quantified CpG, DNA methylation and nucleosome occupancy levels surrounding internal exon and start and stop sites outside of CpG islands for 53404 exons.

perl get_individual_exon_data.pl nomeseq_imr90_hcg/ nomeseq_imr90_gch/ hg18/ hg18_internal_exon_noCpGIs_data.txt

55. determined the average CpG, mHCG/HCG, and uGCH/GCH fractions relative to internal exon and start and stop sites outside of CpG islands

perl get_average_exon_data.pl individual_exon_start_hcg_all.txt individual_exon_start_hcg_meth.txt individual_exon_start_gch_all.txt individual_exon_start_gch_meth.txt individual_exon_start_cg_all.txt individual_exon_stop_hcg_all.txt individual_exon_stop_hcg_meth.txt individual_exon_stop_gch_all.txt individual_exon_stop_gch_meth.txt individual_exon_stop_cg_all.txt

Primary Output Files: exon_start_data_cg_meth_oc.txt, exon_stop_data_cg_meth_oc.txt

56. used R codes and a little photoshop to produce figure S12

Rscript figureS12.exonstart.code.R

Rscript figureS12.exonstop.code.R

Primary Output Files: figureS12.exonstart.jpg, figureS12.exonstop.jpg

57. determined the average CpG, mHCG/HCG, and uGCH/GCH fractions for each nucleosome in all features

perl feature_nucleosome_definer.pl nomeseq_imr90_hcg_chr1.txt nomeseq_imr90_gch_chr1.txt hg18/chr1.fa IMR.90.annotated_reads /annotated_IMR90_MNase-seq_chr1.txt hg18_CpG_Islands_filtered.txt chr1

replicated for all chromosomes

58. generated boxplot data and made figureS13

perl getBoxPlotData.feature.mcpg.oc.cpgi.pl FigureS13/

perl getBoxPlotData.feature.mcpg.oc.pl FigureS13/

Rscript figureS13.code.R

Primary Output Folder: FigureS13

Primary Output File: figureS13.jpg

6) **Figures 5, S14, and S15 - DNA methylation and Nucleosome Occupancy in domains marked by postranslationally marked chromatin**
A. **Figure S14 and ChIP-seq data processing**
59. downloaded processed IMR90 RoadMap ChIP-seq data in BED format with hg19 coordinates, reformatted data to into a workable BED format (download commands not given) and called peaks with SICER

sh reformat.sh

cd HM

perl multiSICER.pl ./

60. converted SICER peaks to hg18 coordinates with UCSC liftover tool and sorted converted peaks

cp */Results/*/* X-W200-G600-FDR0.01-island.bed ./

perl makeLiftoverShellScript.pl ./

sh liftover.sh

mkdir hm.sorted

perl sortBED.pl ./

61. used bedTools multi-intersect bed command

perl makeMultiIntersectBedCommand.pl hm.sorted/

sh multiIntersectBed.sh

62. annotated every base pair in SICER peaks and generated figureS14

mkdir byBase

perl getHMbyBase.pl histone_modification_combination_domains.txt byBase/

mkdir AnnotatedByBase

mkdir AnnotatedByBaseStats

perl makeMultipleAnnotatePeaksJobs.pl byBase/ AnnotatedByBase/ AnnotatedByBaseStats/

sh ap_H3K4me3_chr1_byBase.sh

replicated for all modifications and chromosomes

perl combineMultipleAnnotationsByBase.pl AnnotatedByBaseStats/

filtered out undesired features

perl makeMatrix.pl combined_annotations_byBase.txt

used “hm.feature.enrichment.filtered.matrix.txt” to make FigureS14 in Excel

B. **Figure 5A-D**
63. quantified CpG, DNA methylation and nucleosome occupancy levels surrounding in histone modification domains relative to MNase-seq derived nucleosome midpoints

mkdir hmProfiles

perl analyzeHMandNOMeSeq3.pl nomeseq_imr90_hcg_chr1.txt nomeseq_imr90_gch_chr1.txt hg18/chr1.fa reads/IMR90_MNase-seq_chr1.bed histone_modification_combination_domains.txt chr1

replicated for all chromosomes

64. determined the average CpG, mHCG/HCG, and uGCH/GCH fractions in histone modification domains relative to MNase-seq derived nucleosome midpoints

perl getHMprofiles.pl hmProfiles/

65. computed the average mCpG and uGCH/GCH fractions in 123 bp central region of nucleosomal DNA in histone modification domains relative to MNase-seq derived nucleosome midpoints

perl get.NucOp.mCpG.HM.correlationData.pl single_histone_methylation_profiles_GCH.txt single_histone_methylation_profiles_HCG.txt single_histone_methylation_profiles_CG.txt

66. used R codes and a little photoshop to generate Figure 5A-D

Rscript figure5ABCD.code.R

Rscript figure5D.legend.code.R

C. **Figure 5E-G and Figure S15**
67. used Yaping Lui’s unpublished scripts to determine Z-scores from genome-wide ChIPseq data

mkdir wig

sh bed2bw.sh

sh makeWig.sh

68. combined chromosomal nucleosome data files from figure 3 into a single bed6 formatted file and converted the data from hg18 to hg19 coordinates

perl getBED.pl NOMeSeq.individual.nucleosome.no.CpGI.info /

sh liftover2.sh

69. determined average DNA methylation, nucleosome occupancy, CpG, and mCpG per nucleosome values for single and paired histone modifications weighted by Zscores.

perl HMpairZscore.pl wig/ imr90.hg19.MNase-seq.no.meth.cg.bed

perl single matrix.pl histone_modification_summary_data_genome.txt

70. made figures 5E, 5F, S15A, S15B, and S15D in R studio with code in text file below

figure5EFS15AB.Rcode.txt

figureS15D.Rcode.txt

71. obtained data for Figure 5G and S15C, generated figure, and assembled Figure 5 panels in photoshop

mkdir Figure5G

cp *genome.txt Figure5G/

perl getHMCorrelationData.pl Figure5G/

figure5GS15C.Rcode.txt

7) **Figures 6, S16, and S17 - DNA methylation and Nucleosome Occupancy in CpG Islands**
A. **Figure 6A**
72. computed the number of CpGs, G+C content, average mHCG/HCG ratio, average uGCH/GCH ratio for each nucleosome inside CpG islands between positions -61 and +61 relative to the MNase-seq derived nucleosome midpoint

perl nucleosome_definer_cpgi.pl ../nomeseq_imr90_hcg_chr1.txt ../nomeseq_imr90_gch_chr1.txt ../hg18/chr1.fa ../IMR.90.reads/IMR90_MNase-seq_chr1.txt ../hg18_CpG_Islands.txt chr1

replicated for all chromosomes

73. generated boxplot data for CpG island nucleosomes

perl makeBoxPlotData.cpgi.pl NOMeSeq.individual.nucleosome.CpGI.info/

74. used R code to produce figure 6A

Rscript figure6A.code.R

B. **Figures 6B and 6C**
75. divided CpG islands into nonTSS and TSS

perl getCpGITSSoverlap.pl hg18_CpG_Islands.txt hg18_RefSeq_Genes_Filtered_sorted_removed_duplicates.txt

76. defined nonTSS and TSS CpG island nucleosomes

perl nucleosome_definer_cpgi_tss_500.pl ../nomeseq_imr90_hcg_chr1.txt ../nomeseq_imr90_gch_chr1.txt ../hg18/chr1.fa ../IMR.90.reads/IMR90_MNase-seq_chr1.txt cpgi_tss_500.txt chr1

replicated for all chromosomes

perl nucleosome_definer_cpgi_non_500.pl ../nomeseq_imr90_hcg_chr1.txt ../nomeseq_imr90_gch_chr1.txt ../hg18/chr1.fa ../IMR.90.reads/IMR90_MNase-seq_chr1.txt cpgi_non_500.txt chr1

replicated for all chromosomes

77. generated matrix data for figures 6B and 6C and generated figures in R studio with code in text file below

perl makeTssCpGImatrix.pl NOMeSeq.individual.nucleosome.CpGI.TSS.info/

perl makeNonTssCpGImatrix.pl NOMeSeq.individual.nucleosome.CpGI.nonTSS.info/

figure6BC.Rcode.txt

C. **Figures 6D, 6E, S16, and S17**
78. used a combination of the perl script below as well as the HOMER annotatePeaks function to ultimately generate TSS, genebody, and intergenic CpG island coordinate files in BED format

perl getCpGITSS500coordinatesForFigure6D.pl hg18_CpG_Islands.txt hg18_RefSeq_Genes_Filtered_sorted_removed_duplicates.txt

79. aligned NOMe-seq data to CpG islands with Yaping Lui’s perl script

sh wigToBigWig.sh

sh align.hcg.gch.hg18.sh

80. sorted NOMe-seq data by decreasing nucleosome occupancy for 3 CpG island types and made CDT files using 101 21 base pair bins for Java TreeView input

perl getFigure6DEorderNOMeSeqCDT.pl

CPGI.gch.alignedTo.CPGI-tss.2700.txt

CPGI.gch.alignedTo.CPGI-genebody.2700.txt	CPGI.gch.alignedTo.CPGI-intergenic.2700.txt	CPGI.hcg.alignedTo.CPGI-tss.2700.txt	CPGI.hcg.alignedTo.CPGI-genebody.2700.txt	CPGI.hcg.alignedTo.CPGI-intergenic.2700.txt

81. generated BED file corresponding to order of CpG island coordinates in from Step 68, converted bed file to hg19 coordinates, and aligned ChiP-seq Z-score data to hg19 CpG island coordinates with Yaping Lui’s script

perl getBEDfromCDT.pl gch.TSS.genebody.intergenic.ordered.cdt

used UCSC genome browser liftover tool on website to convert to CpG Island bed file to hg19 coordinates

sh align.chipseq.hg19.sh

82. made CDT files using 101 21 base pair bins, combined 15 ChiPseq datasets into one large CDT file for Java TreeView input.

perl getBEDfromCDT.pl gch.TSS.genebody.intergenic.ordered.cdt

used UCSC genome browser liftover tool on website to convert to CpG Island bed file to hg19 coordinates

sh align.chipseq.hg19.sh

perl getMulti.21.1001.pl ./

perl getBigCDT.pl ./

83. reordered CDT files by importing as dataframes in R and sorting by decreasing H3K27me3 and H3K9me3 signals for figures S16 and S17, respectively, and subsequently, used Java TreeView to make the heatmaps.
8) **Figures S19 - DNA methylation and Nucleosome Occupancy in Partially Methylated Domains**
84. downloaded IMR90 partially methylated domain coordinates and quantified CpG, DNA methylation and nucleosome occupancy levels surrounding in histone modification domains relative to MNase-seq derived nucleosome midpoints

perl NOMeSeq.pmd.pl ../nomeseq_imr90_hcg_chr1.txt ../nomeseq_imr90_gch_chr1.txt ../hg18/chr1.fa reads/IMR90_MNase-seq_chr1.bed imr90.pmd.bed chr1

replicated for all chromosomes

85. determined the average CpG, mHCG/HCG, and uGCH/GCH fractions in histone modification domains relative to MNase-seq derived nucleosome midpoints

perl getNOMeSeqPMDCGprofiles.pl NOMeSeq_PMD/

perl getPMDHCG.pl NOMeSeq_PMD/

perl getPMDHCG.pl NOMeSeq_PMD/

perl combinePMD.pl nomeseq_PMD_GCH.txt nomeseq_PMD_HCG.txt nomeseq_PMD_CG.txt

86. determined histone modification peak enrichment in PMDs

sh intersectBed.sh

perl getOverlap.pl PMD/

perl getHistogramData.pl overlap.txt

87. used R codes and a little photoshop to generate Figure S19A-D

Rscript figureS19ABC.code.R

Rscript figureS19D.code.R

9) **Figures 7 and S18 - DNA methylation and Nucleosome Occupancy at Conserved Transcription Factor Binding Sites**
A. **Figures 7A and 7B**
88. downloaded HCT116 NOMe-seq data and WGBS in bigWig and BED formats, respectively, from GEO for Figures 7 and S16, downloaded HCT116 ChIP-seq and DNase-seq data for Figure 7 from ENCODE, downloaded HCT116 ChIP-seq data for Figure S16 from GEO, and downloaded conserved transcription factor binding sites from the UCSC genome browser

cd ..

mkdir HCT116

cd HCT116

sh getEncode.sh (only commands for downloading ENCODE TF data are in this script)

89. determined DNA methylation status for each conserved CpG-containing transcription factor binding site and divided them into unmethylated and methylated groups

perl convertBEDtoWIG.pl GSM1465024_ResultCount_C166TACXX_1_WIT1251A53.hg19_rCRSchrm.fa.realign.mdups.recal.cpg.filtered.sort.CG.6plus2.bed

perl getTFBSmethWGBS.pl hct116.hg19.wgbs.percent.wig hg19_rCRSchrm.fa tfbsConsSites.hg19.txt

perl getTFBSmuWGBS.pl tfbsConsSites.hg19.ucsc.motif.cg.meth.WGBS.txt

90. aligned NOMe-seq and WGBS data to TFBSs with Yaping Lui’s perl script, sorted alignments by decreasing nucleosome occupancy, generated CDT files, and extracted TFBS order

wigToBigWig hct116.hg19.wgbs.percent.wig hg19.chrom.sizes hct116.hg19.wgbs.bw

sh align.NOMeSeq.WGBS.sh

perl getFigure7AorderNOMeSeqWGBSCDT.pl tfbs.u.WGBS.bed tfbs.m.WGBS.bed TFBS.gch.alignedTo.Unmethylated_WGBS_TFBS.2700.txt TFBS.gch.alignedTo.Methylated_WGBS_TFBS.2700.txt

TFBS.hct116.alignedTo.Unmethylated_WGBS_TFBS.2700.txt

TFBS.hct116.alignedTo.Methylated_WGBS_TFBS.2700.txt

perl getOrderedBed.pl tfbs.u.WGBS.bed tfbs.m.WGBS.bed gch.uTFBS.mTFBS.ordered.wgbs.cdt

91. align ENCODE H3K27ac and DNase signals to TFBSs using NGSplot

perl convertToNGSplotBED.pl tfbs.u.m.ordered.WGBS.bed tfbs.u.m.ordered.WGBS.same.bed

sh H3K27ac.DNase.sh

92. generated CDT files for H3K27ac and DNase-seq data for Figure 7A

getTextFromRdata.Figure7A.txt

perl getDNaseH3K27acCDT.pl umDNase.ordered.wgbs.txt umH3K27ac.ordered.wgbs.txt

93. generated data for metaplots in Figure 7B and data from Figures 7A and 7B using Java TreeView and Rcode.

perl getMetaPlot2700.pl TFBS.gch.alignedTo.Unmethylated_WGBS_TFBS.2700.txt TFBS.gch.alignedTo.Methylated_WGBS_TFBS.2700.txt

Rscript figure7B.code.R

B. **Figures 7C and 7D**
94. aligned transcription factor occupancy data to methylated and unmethylated of 10 conserved CpG-containing transcription factor binding sites

perl getFactor.pl tfbs.u.m.ordered.WGBS.bed

sh ngsplot.commands.sh

sh unzip.sh

95. assembled unmethylated and methylated metaplot occupancy data in large spreadsheet called “hct116.encode.factor.chipseq.m.u.avg.WGBS.txt” and use Rcode to generate Figure 7D

Rscript figure7D.code.R

96. generated heatmap data for Figure 7C and plotted data using Java TreeView

getTextFromRdata.SP1Rdata.txt

cat uSP1.txt mSP1.txt > umSP1.txt

convert umSP1.txt to CDT file

perl make.SP1.CDT.pl gch.uTFBS.mTFBS.ordered.wgbs.cdt wgbs.uTFBS.mTFBS.ordered.wgbs.cdt umDNase.ordered.wgbs.cdt umH3K27ac.ordered.wgbs.cdt umSP1.cdt

C. **Figure S18**
97. downloaded data from GEO
98. generated heatmap data for histone modifications and CpG island presence for figure S18 and used Java TreeView for visualization

sh align.Jones.sh

perl getCpGIanno.pl tfbs.u.m.ordered.WGBS.bed hg19.UCSC.CpGIs.BED6.bed
